# *Exo*-cage catalysis and initiation derived from photo-activating host–guest encapsulation†

**DOI:** 10.1039/d3sc04877b

**Published:** 2023-11-22

**Authors:** Rebecca L. Spicer, Helen M. O'Connor, Yael Ben-Tal, Hang Zhou, Patrick J. Boaler, Fraser C. Milne, Euan K. Brechin, Guy. C. Lloyd-Jones, Paul J. Lusby

**Affiliations:** a EaStCHEM School of Chemistry, University of Edinburgh Joseph Black Building, David Brewster Road Edinburgh Scotland EH9 3FJ UK Paul.Lusby@ed.ac.uk Guy.Lloyd-Jones@ed.ac.uk E.Brechin@ed.ac.uk

## Abstract

Coordination cage catalysis has commonly relied on the endogenous binding of substrates, exploiting the cavity microenvironment and spatial constraints to engender increased reactivity or interesting selectivity. Nonetheless, there are issues with this approach, such as the frequent occurrence of product inhibition or the limited applicability to a wide range of substrates and reactions. Here we describe a strategy in which the cage acts as an exogenous catalyst, wherein reactants, intermediates and products remain unbound throughout the course of the catalytic cycle. Instead, the cage is used to alter the properties of a cofactor guest, which then transfers reactivity to the bulk-phase. We have exemplified this approach using photocatalysis, showing that a photoactivated host–guest complex can mediate [4 + 2] cycloadditions and the aza-Henry reaction. Detailed *in situ* photolysis experiments show that the cage can both act as a photo-initiator and as an on-cycle catalyst where the quantum yield is less than unity.

## Abstract 2

We report a new class of photoredox catalysis arising from the non-covalent association of separately inactive coordination cage and guest.

## Graphical abstract

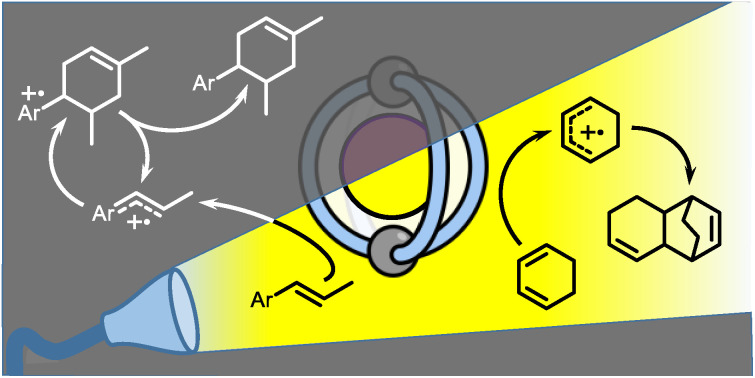

## Introduction

**Fig. 1:**
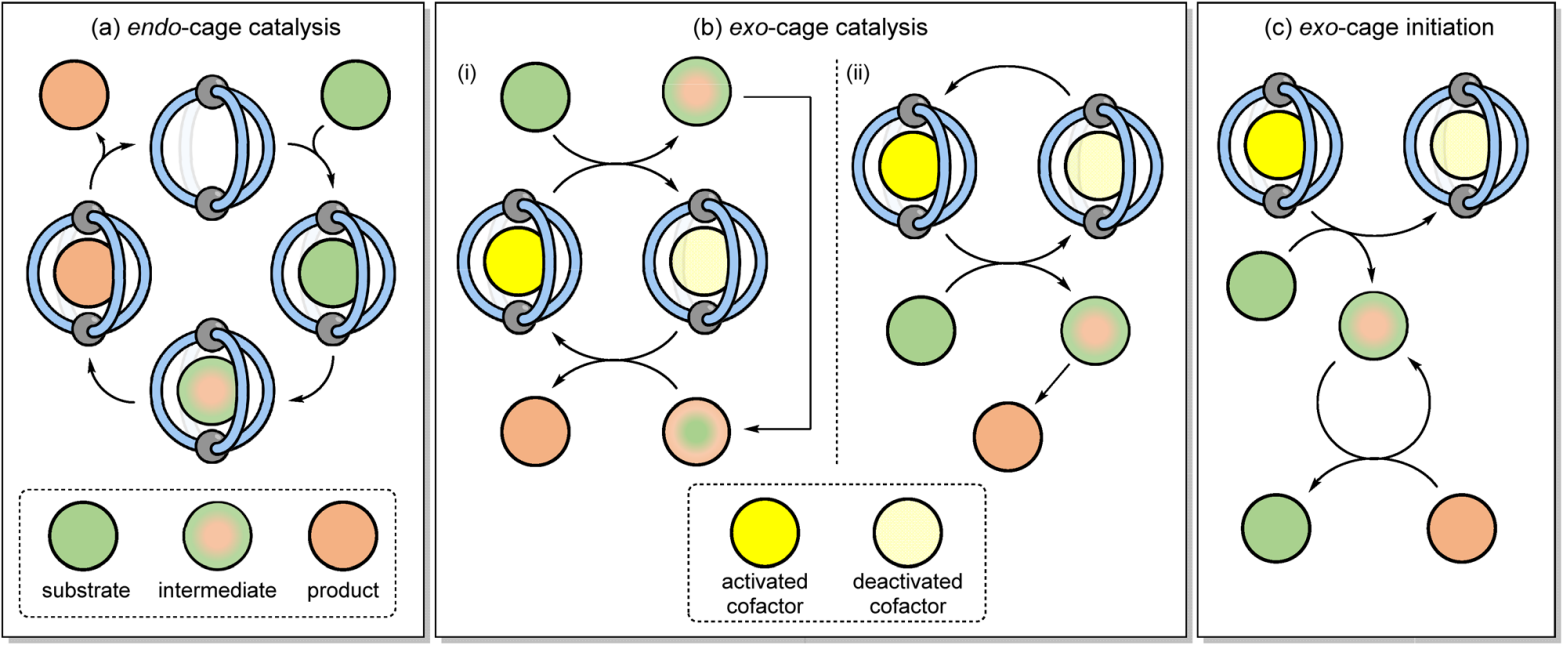
(a) *Endo*-cage catalysis involves the binding of substrates, intermediates and products whereas (b) *exo*-cage catalysis and (c) *exo*-cage initiation involves bond breaking and/or formation in the bulk phase, triggered by the release of reactivity from a cage-cofactor complex.

Coordination cages have emerged as a class of self-assembled molecular–hosts that can be utilised in areas ranging from separation^1–3^ and drug delivery^4–7^ through to catalysis.^8–13^ In applications such as separation or drug delivery, function derives solely from “passive” binding *i.e.*, there is no requirement for the host to modulate the properties of the encapsulated species. The host–guest chemistry involved in catalysis is more nuanced because the bound substrate(s) must in some way possess enhanced reactivity compared to the non-bound species. This increased chemical reactivity can either derive from binding in a specific conformation^14^ or by being co-encapsulated with another species,^8^ in both instances leading to a reduction in the entropy of activation. Alternatively, the binding can induce some physiochemical change (*e.g.*, altering p*K*_a_ or redox potential),^9,15–17^ leading to an increased rate of reaction that originates from enthalpic effects. As the bound species is constitutionally dynamic (Fig. 1a) *i.e.*, it changes from a reactant to a product *via* intermediates and transition states (TS), catalytic function hinges on how well the cage differentiates these different species; over-stabilisation of the substrate can lead to poor activity while high affinity towards the product will lead to inhibition. The complexities of being able to bind different species with varying affinities perhaps explains why only a small fraction of the many hundreds of reported cages are known to be effective catalysts.

An alternative to the conventional strategy of binding the substrate, intermediate and product in a catalytic cycle (Fig. 1a) is to use the cage as an exogenous catalyst (Fig. 1b).^18–20^ With this strategy, reactivity is generated through the collisional quenching of an activated cage-cofactor host–guest complex with the substrate. The subsequent non-bound reactive intermediate then undergoes bond-breaking/forming steps away from the supramolecular complex. This approach can be viewed as complementary to conventional confined reactivity; the separation of the host from the reactive intermediate(s) will limit the opportunity for the cage to induce unusual selectivity, yet an exogenous catalyst would possess a wider and more applicable substrate scope. Crucially, problems such as product inhibition would be sidestepped. This would also allow lower loadings of cage complex to be used than is conventionally the case.

There are several mechanisms by which reactivity could be released from a cofactor-cage complex into the bulk. For example, electrons could be relayed from the ground state host guest complex to or from a free substrate *via* outer sphere electron transfer (ET), and finally photoexcitation of the host–guest complex would allow reactivity to be released *via* either energy transfer or ET mechanisms. The *in situ* generation of bulk-phase reactivity using the host–guest complexation of otherwise benign starting materials also provides other benefits such as removing the need to handle sensitive (*e.g.*, photodegradable) chemicals.

The definition “*exo*-cage catalysis” also indicates that the cage-cofactor complex is part of a catalytic cycle. This would be the case, for example, if the reactivity that is “released” from the cage-cofactor complex to generate a reactive intermediate is then “returned” at some stage during product formation (Fig. 1(b)i). Alternatively, the reactivity could rapidly dissipate upon the formation of the product such that external cage-co-factor regeneration is required (Fig. 1(b)ii). It is also feasible that the cage-co-factor complex can sub-stoichiometrically promote the reaction, without acting as a catalyst. In this mode, a reactive intermediate is generated outside of the cage, and then this non-cage species sustains a chain reaction mechanism (Fig. 1(c)). This would negate the need to restore the active cage-cofactor complex. Herein, we describe how coordination cages can be used to mediate a series of different reactions through apparent catalytic and initiation mechanisms that rely on the host–guest complexation to generate *exo*-cage reactivity based on photoexcitation.

## Results and discussion

**Scheme 1:**
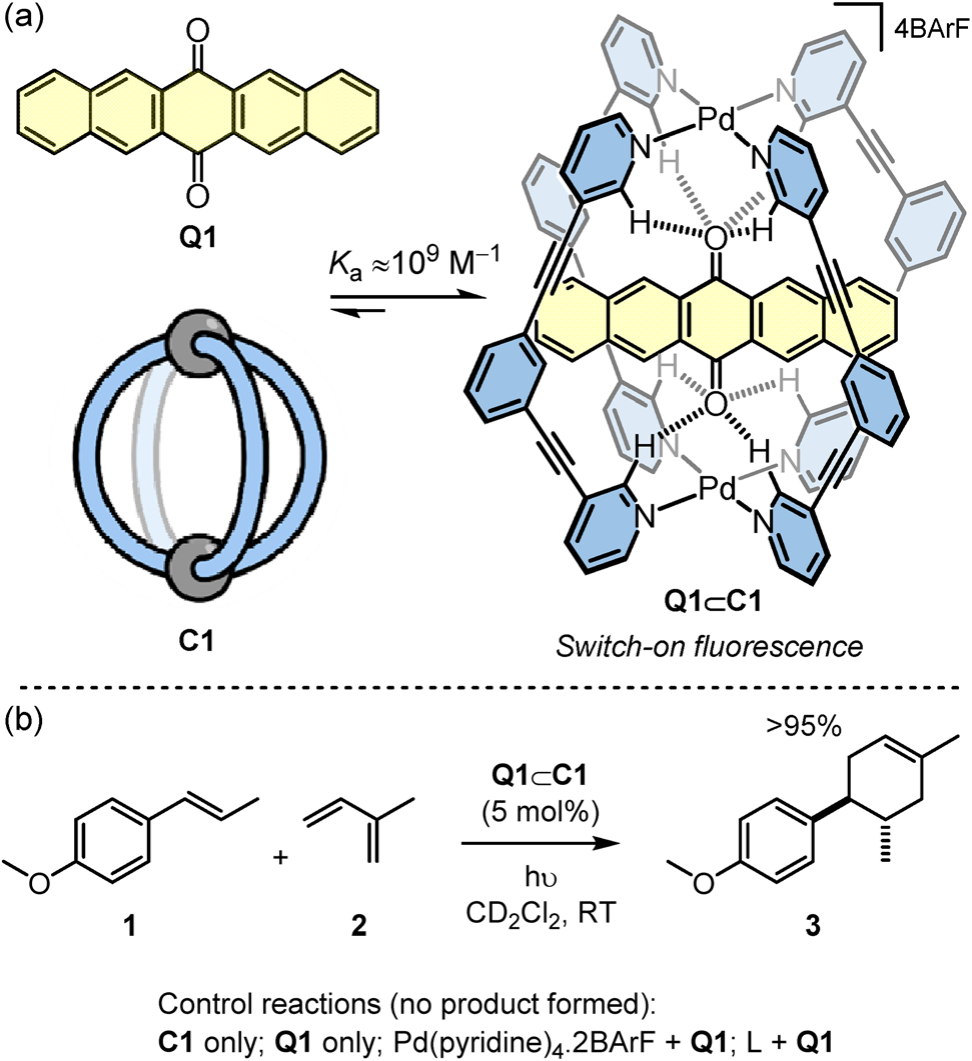
(a) The Q1⊂C1 photocatalyst is generated through high-affinity complexation of host and guest that are separately inactive. (b) Q1⊂C1 photocatalysed [4 + 2] reaction of 1 with 2 to generate 3.

The cage used in this study is the simple Pd_2_L_4_ cage C1 that was originally reported by Hooley (Scheme 1a).^21^ This cage binds quinones because the inward facing *ortho*-pyridyl hydrogen atoms, which are rendered H-bond acidic due to proximity to the Pd^2+^ ions, are able to simultaneous interact with both oxygen atoms of the guest. In the case of the extended quinone, Q1, the guest is encapsulated with a very high affinity (*K*_a_ ≈ 10^9^ M^−1^).^22^ Furthermore, the binding causes switch-on fluorescence of Q1. Combining this photoactivation with the known capacity of the cage to enhance the redox properties of bound quinones^19^ suggested that Q1⊂C1 may be an effective photoredox catalyst. This compound is held together by only weak non-covalent interactions making it distinct from the most popular class of photocatalysts that include mononuclear transition metal complexes^23–26^ and organic photoactive compounds.^27–29^

The [4 + 2] cyclisation of *trans*-anethole, 1, with isoprene, 2, to give 3 was selected as an initial benchmark reaction to investigate the photocatalytic properties of Q1⊂C1 (Scheme 1b). This reaction is well studied, having been shown to be promoted by redox initiators^30^ and photoactive compounds. In particular, Yoon has carried out several detailed studies using the irradiation of archetypal photoactive Ru tris(chelate) metal complexes, such as Ru(bpy)_3_^2+^ (bpy = 2,2′-bipyridine) and the more active Ru(bpz)_3_^2+^ (bpz = bipyrazine).^25,31,32^

When a sample of 1, 2 and Q1⊂C1 in CD_2_Cl_2_ under ambient light was monitored by ^1^H NMR spectroscopy, quantitative formation of 3 was observed after 24 hours. In contrast, the reactions using C1 only or Q1 only in place of Q1⊂C1 showed no product (see Fig. S2†). The dependence of the reaction on light was also ascertained by repeating the Q1⊂C1 reaction in an amberised NMR tube (see Fig. S2†). This showed only starting materials, indicating that the reactivity of the host–guest complex stems from photoexcitation.

To increase the reaction rate, the ambient lighting was changed to an LED source (Table 1). Using 530 nm green irradiation, 5 mol% Q1⊂C1 with respect to 1, and a large excess of 2 (40 eq.), irradiation for one hour gave close to quantitative yield of 3 (Table 1, entry 1). Considering that Q1⊂C1 shows negligible absorbance at 530 nm (see Fig. S26†), we switched to a blue 460 nm LED where the host–guest complex does possess an absorbance band. At this shorter wavelength, the reaction time can be reduced to just 5 seconds without affecting the yield (Table 1, entry 2). The scale of the reaction can also be increased, and the loading of catalyst dropped, albeit with slightly longer reactions times (Table 1, entries 3–5). Using optimised conditions we were able to isolate 66 mg of product 3 in 90% yield (Table 1, entry 5), showing the feasibility of a preparative scale cage-catalysed reaction. Additional controls under LED irradiation have also been undertaken (Fig. S5†), which include replacing Q1⊂C1 by (i) Q1 plus the representative mononuclear complex, Pd(pyridine)_2_·2BArF and (ii) Q1 plus the free ligand, L. In neither case was any conversion of 1 + 2 to 3 detected.

**Table 1:** Q1⊂C1 photocatalysed reaction of 1 with 2 to generate 3^a^

Entry	Mol% Q1⊂C1	LED wavelength (nm)	Time	Yield^b^3 (%)
1	5	530	1 h	>95
2	5	460	5 s	>95
3^c^	1	460	10 min	>95
4^c^	0.5	460	10 min	>95
5^d^	1	460	20 min	90^e^

a Standard conditions: RT, *hν*, Q1⊂C1 (0.5 mM), 1 (10 mM), 2 (400 mM), CD_2_Cl_2_ (625 μL).

b Yields determined by ^1^H NMR spectroscopy (error estimated to be ±5%).

c CH_2_Cl_2_ (3.7 mL), Q1⊂C1 (0.1–0.2 mM), 1 (19 mM), 2 (760 mM).

d CH_2_Cl_2_ (4.6 mL), Q1⊂C1 (0.7 mM), 1 (74 mM), 2 (2.9 M).

e Isolated yield.

Cyclohexadiene, 4, is a substrate that can undergo light-induced [4 + 2] cycloaddition with itself to give the unsymmetrical homodimer 5. Ferreira has reported that Ru(bpz)_3_^2+^ is poorly effective at promoting this cycloaddition reaction, finding that the more strongly oxidising Cr(dmcbpy)_3_^3+^ (dmcbpy = 4,4′-dimethylcarboxylate-2,2′-bipyridine, *E*_1/2_* = +1.84 V *vs.* SCE) gives much higher yields.^33^ It is suggested that this is consistent with the relatively high oxidation potential of 4 (*E*(4) = +1.53 V *vs.* SCE, CH_3_CN, irreversible *c.f.*, *E*(1) = +1.11 V).^34^

Full intensity 460 nm LED irradiation of a CD_2_Cl_2_ solution of 4 with 5 mol% Q1⊂C1 for 2 minutes gave product 5 in 60% yield, with close to 90% substrate consumption (Table 2, entry 1). This product yield is very similar to what Ferreira reports (66%) using Cr(dmcbpy)_3_^3+^, albeit the chromium-complex mediated reaction uses 2 mol% catalyst loading but with shorter wavelength near-UV irradiation. Ferreira's investigation also showed that better yields were obtained in more polar solvents, such as nitromethane. In contrast, the generation of 5 with Q1⊂C1 was much less effective in polar solvents such as nitromethane and acetonitrile (Table 2, entries 2 and 3). It is important to note that this lower reactivity is not due to the displacement of Q1 by disruption of the non-covalent host–guest interactions: the slow rates of guest exchange in Q1⊂C1 allow ^1^H NMR spectroscopic detection of this species throughout these reactions (see Fig. S33 and S34†). The addition of different counteranions was also briefly investigated, which we reasoned may influence the stability of any reactive radical-cation intermediates (see below). In this experiment, we added just half an equivalent of NBu_4_PF_6_ with respect to Q1⊂C1 (Table 2, entry 4) to avoid precipitation of the tetra-PF_6_ cage complex. Again, this showed no improvement in yield of 5 – which similarly cannot be explained by guest displacement, as the host–guest complex remains stable under the reaction conditions (see Fig. S35†). Finally, we looked at the effect of introducing air, which has been shown to have a significant effect on the chromium-promoted reaction.^35^ In order to probe this, we carried out the reaction in a microwave vial instead of within a sealed NMR tube, so that the reaction could be continually sparged with air during irradiation. The yield of 5 under these conditions (78%; Table 2, entry 5) exceeds that previously reported by Ferreira (66%), highlighting the efficacy of the host–guest photocatalytic system. While the exact mechanism by which air affects this reaction has not been investigated, it is possible that oxygen mediates energy and/or electron transfer between the various states of host–guest complex and the substrate and/or any radical cation intermediates.^35^

**Table 2:** Q1⊂C1 photocatalysed reaction of 4 to generate 5^a^

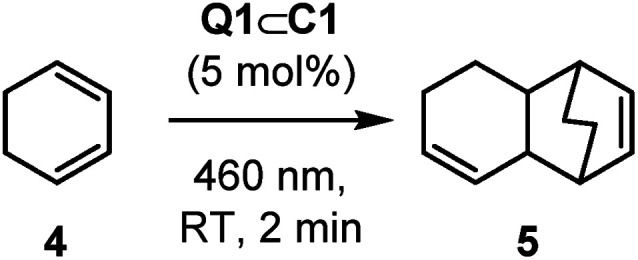			
Entry	Solvent	Additive	Yield^b^5 (%)
1	CD_2_Cl_2_	—	60 (90^c^)
2	CD_3_NO_2_	—	14
3	CD_3_CN	—	3
4	CD_2_Cl_2_	NBu_4_PF_6_^d^	50
5	CD_2_Cl_2_	Air^e^	78

a Standard conditions: RT, 460 nm LED, Q1⊂C1 (0.5 mM), 4 (10 mM), solvent (575 μL).

b Yields determined by ^1^H NMR spectroscopy (error estimated to be ±5%).

c % consumption 4.

d 2.5 mol% NBu_4_PF_6_.

e Q1⊂C1 (0.5 mM), 4 (10 mM), solvent (3 mL) in a microwave vial continually sparged with a stream of air.

We further studied the photoactivity of Q1⊂C1 using an *in situ* irradiation NMR technique (see ESI, Section 3.6).† This method uses a 455 nm LED coupled to an optical fibre with a light-diffusing tip, which is located within a quartz insert immersed in the reaction mixture in a 5 mm NMR tube.^36,37^ In initial experiments, a series of eight identical reactions of 1 with 2 were analysed, with each receiving a train of NMR pulses, separated by 5 seconds. The first pulse was applied after a pre-defined irradiation time, which was incremented through the series using precise electronic timing of both the LED and the spectrometer. The resulting data were interleaved to provide a pseudo-temporal concentration profile, the rate being in accordance with the *ex situ* experiment (Table 1, entry 2). To facilitate more detailed analysis, the catalyst loading and the LED power were reduced until kinetic data could be collected by *in situ* NMR spectroscopy in real time, rather than by interleaving. Systematic variations in the LED power, and the initial catalyst concentration, revealed linear dependencies on both (Fig. S12 and S13†).

**Fig. 2:**
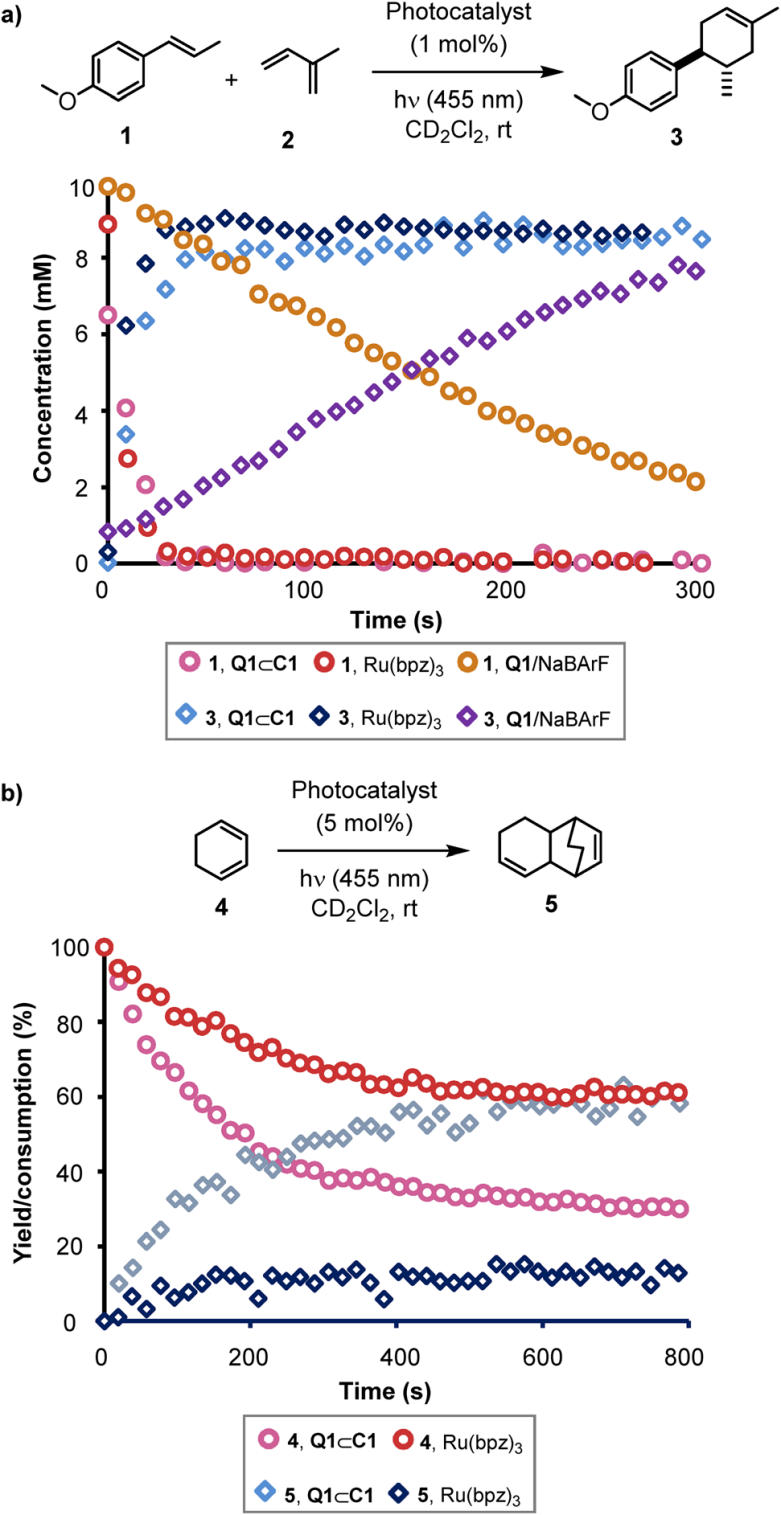
Kinetic data obtained from *in situ* NMR-LED experiments. (a) A comparison of the Q1⊂C1, Ru(bpz)_3_(BArF)_2_ and Q1/NaBArF photo-catalysed reaction of 1 + 2 → 3 under otherwise identical conditions using 1 mol% catalysts and 5% LED power (455 nm). (b) The photo-catalysed reaction of 4 → 5, using either Q1⊂C1 or Ru(bpz)_3_(BArF)_2_. Conditions as shown in Table 2, entry 1, with irradiation at 455 nm using 5% LED power.

Using the *in situ* NMR spectroscopic method, we sought to make a direct comparison of the activity of Q1⊂C1 with Ru(bpz)_3_^2+^ (Fig. 2).^25^ Initial reactions of 1 with 2, using Ru(bpz)_3_(BArF)_2_ to eliminate counteranion effects,^31^ appeared to indicate that the host–guest system is an even more active catalyst than the conventional mononuclear transition metal complex for this specific cycloaddition. However, during further experiments to allow comparison of the quantum yield using Ru(bpz)_3_(BArF)_2_, it was observed that the use of different batches of both cage C1 and Ru(bpz)_3_(BArF)_2_ yielded inconsistent kinetics. Further investigation revealed a dramatic, and consistent, rate increase in both cases upon saturation of the reaction solution with NaBArF, and the kinetic inconsistency was attributed to variation in the residual NaBArF co-crystalized with the catalyst during the anion metathesis step required to make the BArF^−^ salt of both compounds. Repeating the earlier *ex situ* control reactions in the absence of one or both of C1 or Q1 during *in situ* monitoring reiterated the absence of reactivity without both components. However, use of Q1 in a saturated solution of NaBArF yielded reactivity, albeit with significantly attenuated kinetics compared to the reaction of 1 and 2 using either Q1⊂C1 or Ru(bpz)_3_(BArF)_2_ under identical conditions (Fig. 2a). UV-vis analysis revealed a significant change in the absorption spectrum of Q1 upon mixing with NaBArF. Beer–Lambert analysis predicts a two-fold increase in reactivity of Q1⊂C1 over Q1 alone. The ten-fold increase in reactivity observed instead indicates that encapsulation of Q1 within C1 enhances its photocatalytic properties beyond the increase in absorbance at 455 nm. There could be numerous possible ways in which the cage achieves this, such as by hindering unproductive quenching of the excited state guest, stabilising the subsequent radical-anion through non-covalent interactions, or altering charge re-combination pathways.

Calculation of the reaction quantum yield for the reaction of 1 and 2 (see ESI†) gave a value significantly higher than 1 for both Q1⊂C1 and Ru(bpz)_3_(BArF)_2_. This is indicative of a chain process underpinning the reaction, consistent with the findings of Yoon.^31^ When combined with the observation that the reaction efficiency depends on the quantity of NaBArF present, we propose that in this reaction the BArF^−^ anion acts as an active radical chain mediator, photo-initiated by the host–guest complex.

*In situ* monitoring of the homodimerisation of 4 showed results in line with the initial *ex situ* observations. The reaction stalls below certain loadings (3.75 mol%) of Q1⊂C1 (see Fig. S37 and S38†). Control reactions again indicated a lack of reactivity in the absence of light, Q1, C1 and Q1⊂C1. In line with previous observations,^33^ only a trace amount of product was generated when Ru(bpz)_3_(BArF)_2_ was used as the photocatalyst. Similar results were observed using a mixture of Q1 and NaBArF as the catalyst. In both cases, significantly increasing the reaction time did not improve the yield. The two reactions also show a difference in mass balance; Q1⊂C1 shows good mass balance in the conversion of 4 to 5 whereas Ru(bpz)_3_(BArF)_2_ generates significantly less 5 compared to the amount of 4 consumed. Furthermore, in contrast to the reaction between 1 and 2, addition of NaBArF did not affect the observed kinetics in any way. Measurement of the quantum yield of Q1⊂C1 in this reaction yielded a value of 0.5, with a minimum turnover number (TON) of 8. While not definitive, the quantum yield being less than unity is indicative that in this case Q1⊂C1 is likely acting directly as an on-cycle photocatalyst. The different responses of the two reaction systems to the presence of NaBArF adds credence to this hypothesis.

**Scheme 2:**
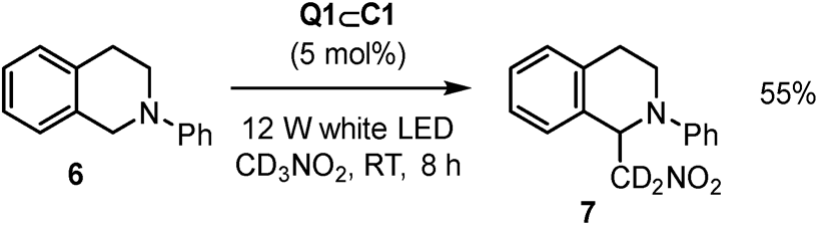
Q1⊂C1 aza-Henry reaction.

The scope of reactions that Q1⊂C1 can promote has also been expanded beyond [4 + 2] cycloaddition to the aza-Henry reaction of 6 to give 7 (Scheme 2).^24^ In this case, Q1⊂C1 appears to be a more modest catalyst (based on yield) compared to representative Ru or Ir transition metal catalysts. As the activity of Q1⊂C1 towards cyclohexadiene dimerisation is attenuated in nitromethane, this could be one possible reason for the less effective aza-Henry catalysis, where nitromethane is both the substrate and the solvent. It is also possible that specific aspects of the aza-Henry mechanism are not well suited to the cage (*e.g.* if charge-recombination is a key step, the protection of the quinone radical-anion by the cage could have a detrimental effect). Nonetheless, it further extends the capability of host–guest complexes to a reaction that has not previously been mediated by this type of supramolecular species.

**Scheme 3:**
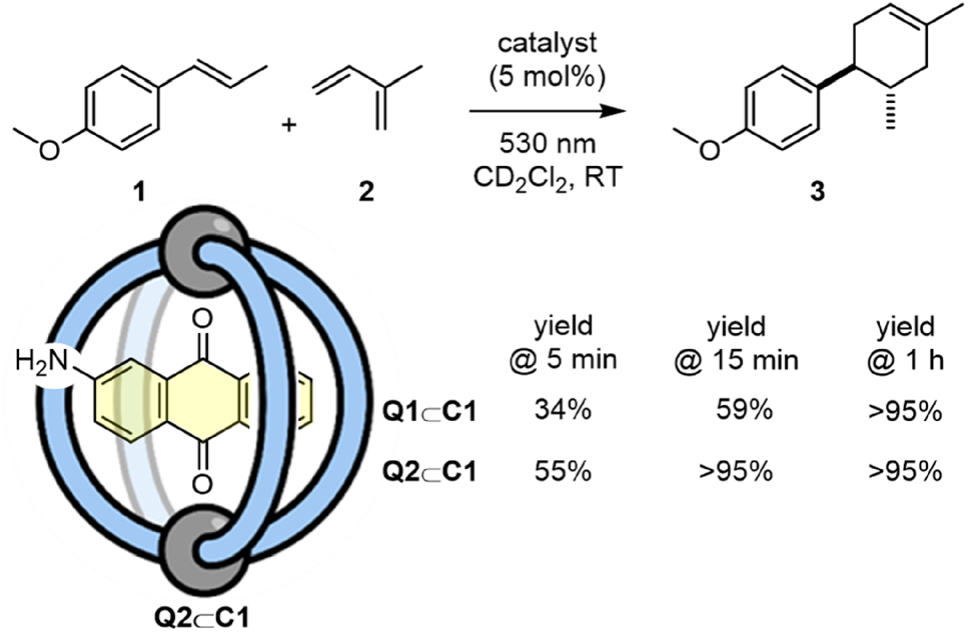
Comparison of catalysts Q1⊂C1 and Q2⊂C1 for the conversion of 1 + 2 → 3 at 530 nm irradiation.

Photocatalysts that are generated by host–guest complexation have the potential to be readily adapted and tuned in a modular fashion by swapping one of the components. In this regard, we have investigated the non-covalent association of aminoanthraquinone, Q2 (Scheme 3). This quinone shows a greater absorption at 530 nm (Fig. S48†), and so it was reasoned that it should be better suited to photocatalysis using a longer wavelength green LED. Indeed, a direct comparison between Q1⊂C1 and Q2⊂C1 for the [4 + 2] cycloaddition of 1 and 2 with irradiation at 530 nm showed that Q2⊂C1 facilitates noticeably shorter reaction times.

## Conclusions

Using the modified reactivity of a bound substrate has served as the dominant approach in supramolecular catalysis since Cramer's pioneering studies with cyclodextrins in the 1960s.^38^ Here we have shown a different strategy, where bond-forming reactions occur away from the supramolecular species but are nonetheless enabled by non-covalent association of host and guest. In this example, we use encapsulation to switch on photocatalytic properties absent in either of the species in isolation. The resultant photoactive host–guest complex shows comparable activity to conventional transition metal complexes that have been widely used in photoredox catalysis.^23–25^ Utilising non-covalent assembly additionally provides a potentially modular way to develop new photocatalysts in which the photophysical and redox properties can be tuned a specific reaction by tailoring both the cage and guest. While the size of cage in this study only allows the encapsulation of a single photoactive species, thus leading to *exo*-catalysis, it may be possible to use a larger host that can additionally bind a substrate, allowing proximity effects and spatial constraints to additionally affect reactivity.^39^ In the future, we also envisage different types of external reactivity (*i.e.*, non-photochemical) could be triggered using encapsulation. As such, we anticipate that the development of systems that are founded using the principles of supramolecular chemistry can continue to contribute to the greater area of catalysis beyond enzyme mimics.^40^

## Author contributions

R. L. S., H. M. O'C., Y. B.-T., H. Z., P. J. B. and F. M. prepared the compounds and/or carried out catalysis reactions. The *in situ* photolysis NMR experiments were conducted by Y. B.-T. All authors aided in the interpretation of the results. P. J. L. conceived the project and wrote the manuscript, with input from all the authors.

## Conflicts of interest

There are no conflicts to declare.

## Supplementary Material

SC-014-D3SC04877B-s001
