# Supplementary material for: Exo-cage catalysis and initiation derived from photo-activating host–guest encapsulation
Source: Chem Sci. 2023 Nov 22;14(48):14140–5. doi: 10.1039/d3sc04877b (PMC10718074; doi:10.1039/d3sc04877b)
Supplement: SC-014-D3SC04877B-s001 [file SC-014-D3SC04877B-s001.pdf]

Electronic Supplementary Material (ESI) for Chemical Science.
This journal is © The Royal Society of Chemistry 2023

Supporting Information

Exo-Cage Catalysis and Initiation derived from Photo-Activating Host-Guest Encapsulation

Rebecca L. Spicer,[a‡] Helen M. O'Connor,[b‡] Yael Ben-Tal,[c‡] Hang Zhou, Patrick J. Boaler, Fraser C. Milne, Euan K. Brechin,* Guy. C. Lloyd-Jones* and Paul J. Lusby*

EaStCHEM School of Chemistry, University of Edinburgh, Joseph Black Building, David Brewster Road, Edinburgh, Scotland EH9 3FJ, UK.

* Paul.Lusby@ed.ac.uk

* Guy.Lloyd-Jones@ed.ac.uk

* E.Brechin@ed.ac.uk

Contents

1 Materials and Methods 4
1.1 *Ex situ* reactions 4
1.2 *In situ* reactions 5
2 Synthesis 5
3 Cage-Quinone Catalyzed *trans*-Anethole and Isoprene Cycloaddition Reactions 6
3.1 Ambient Light 6
3.1.1 General Procedure 6
3.1.2 Product Identification 6
3.1.3 ^{1}H NMR spectra of ambient light catalysed [4+2] reactions 7
3.2 LED Irradiation 8
3.2.1 General Procedure 8
3.3 Catalyst Loading Experiments 10
3.3.1 General Procedure 10
3.3.2 ^{1}H NMR spectra of catalyst loading [4+2] reactions 11
3.4 Preparative Scale Reaction 12
3.5 Quinone Scope 12
3.5.1 ^{1}H NMR spectra of [4+2] quinone scope reactions 13
3.6 *In situ* Illumination NMR Spectroscopy Kinetics Experiments 15
3.6.1 General Procedure 15
3.6.2 Orders in light intensity and catalyst loading 16
3.6.3 On/off studies 17
3.6.4 Measurement of the reaction kinetics at standard catalyst loadings and light intensity 19
3.6.5 Use of different batches of **C1** 21
3.6.6 Comparison to existing photocatalyst $Ru(bpz)_3(BArF)_2$ 21
3.6.7 Addition of NaBArF to the reactions 22
3.6.8 Reaction using only **Q1** and NaBArF 23
3.6.9 ^{1}H NMR spectra of *in situ* irradiated [4+2] cycloadditions 24
3.7 Measuring Radical Chain Length Using LED-NMR 25
3.7.1 Calculating the intensity of the LED-NMR light source with ferrioxolate actinometry 25
3.7.2 Calculating how many incident photons are absorbed by the active catalyst 27
3.7.3 Calculating how much product is produced by the active catalyst 28
3.7.4 Calculation of catalyst quantum yield 29
4 Cage-Quinone Catalyzed 1,3-Cyclohexadiene Cycloaddition Reactions 31
4.1 General Procedure 31
4.1.1 Additive Experiment 31
4.1.2 Aerated Procedure 31
4.2 Product Identification 32

4.3 ^{1}H NMR spectra of [4+2] catalyzed reactions 32
4.3.1 CD_2Cl_2 32
4.3.2 CD_3NO_2 32
4.3.3 CD_3CN 33
4.3.4 CD_2Cl_2 + $NBu_4{\cdot}PF_6$ 33
4.3.5 CD_2Cl_2 + air 34
4.4 *In situ* Kinetic Monitoring with LED-NMR 35
4.4.1 General Procedure 35
4.4.2 Variation of **Q1⊂C1** concentration 35
4.4.3 Variation of light intensity 35
4.4.4 Variation of $Ru(bpz)_3(BArF)_2$ concentration 36
4.4.5 Addition of NaBArF to the reactions 37
4.4.6 Reaction using only **Q1** and NaBArF 38
4.4.7 Calculation of reaction quantum yield, using **Q1⊂C1** as the catalyst 38
4.4.8 Calculation of max TON for cage and Ru catalysed reactions 39
5 Cage-Quinone Catalyzed Aza-Henry Reaction 40
5.1 Procedure 40
5.2 Product Identification 40
5.3 ^{1}H NMR spectra of the aza-Henry reaction 42
6 UV-Vis Experiments 43
6.1 General Procedure 43
6.2 UV/Vis spectra 43
6.2.1 **Q1** 43
6.2.2 **Q2** 43
6.2.3 **Q1** and NaBArF 44
7 References 45

1 Materials and Methods

Unless stated otherwise, all reagents and solvents were purchased from Alfa Aesar, VWR, Fluorochem or Sigma Aldrich and used without further purification. Where the use of anhydrous solvent is stated, drying was carried out using a solvent purification system manufactured by Glass Contour. Column chromatography was carried out using Geduran Si60 (40-63 µm) as the stationary phase and TLC was performed on precoated Kieselgel 60 plates (0.20 mm thick, $60F_{254}$. Merck, Germany) and observed under UV light at 254 nm or 365 nm. All reactions were carried out under air, unless stated otherwise.

Abbreviations used in the Supporting Information include:

RT	Room temperature
NMR	Nuclear Magnetic Resonance
COSY	Correlation Spectroscopy
NOESY	Nuclear Overhauser Effect Spectroscopy
HSQC	Heteronuclear Single Quantum Coherence
HMBC	Heteronuclear Multiple Bond Correlation
MS	Mass spectrometry
HRMS	High resolution mass spectrometry
LED	Light Emitting Diode
TTL	Transistor-transistor logic

Unless stated otherwise, all ^{1}H, ^{13}C and ^{19}F NMR spectra were recorded on either a 500 MHz Bruker AV III equipped with a DCH cryoprobe, a 500 MHz Bruker AV IIIHD equipped with a Prodigy cryoprobe or a 400 MHz Bruker AV III equipped with BBFO+ probe at a constant temperature of 300 K. Chemical shifts are reported in parts per million. Coupling constants (*J*) are reported in hertz (Hz). Standard abbreviations indicating multiplicity were used as follows: m = multiplet, q = quartet, t = triplet, d = doublet, s = singlet, bs = broad singlet. All analysis was performed with MestReNova, Version 12.0.3. All assignments were confirmed using a combination of COSY, NOESY, HMBC and HSQC NMR.

All UV/Vis spectroscopy was carried out on a Shimadzu UV-1900 Spectrophotometer running UV Probe, Version 2.70 (Shimadzu). All data was analyzed and plotted using Origin 2015 software. All measurements were made at room temperature (16–21 °C) in CH_2Cl_2 using a fused silica cuvette with a 10 mm path length, unless stated otherwise.

1.1 *Ex situ* reactions

Aside from Section 5, all *ex situ* irradiated reactions were carried out under either blue (460 nm) or green (530 nm) LED irradiation. The photoreactor (Figure S1a-c) is composed of a 24 V direct current (DC) power supply and a right angle through hole DC power socket, with external cooling provided through the use of a DC axial fan, purchased from RS Components. For the LED lights, the photoreactor is equipped with either LEDST60BL (blue, 460 nm) or LEDST60GN (green, 530 nm) LED strips purchased from Expert Electrical Supplies Ltd. and connected through a plug-in power supply for LED strips. For aerated experiments, a Penn-Plax AirTech 2K0 pump was used to bubble compressed air through the reaction a rate of 0.3 m/s.

For Section 5, the *ex situ* irradiated reactions were carried out under irradiation with a 12 W white LED provided by a TACKLIFE Cree 12 W LED torch (Figure S1d).

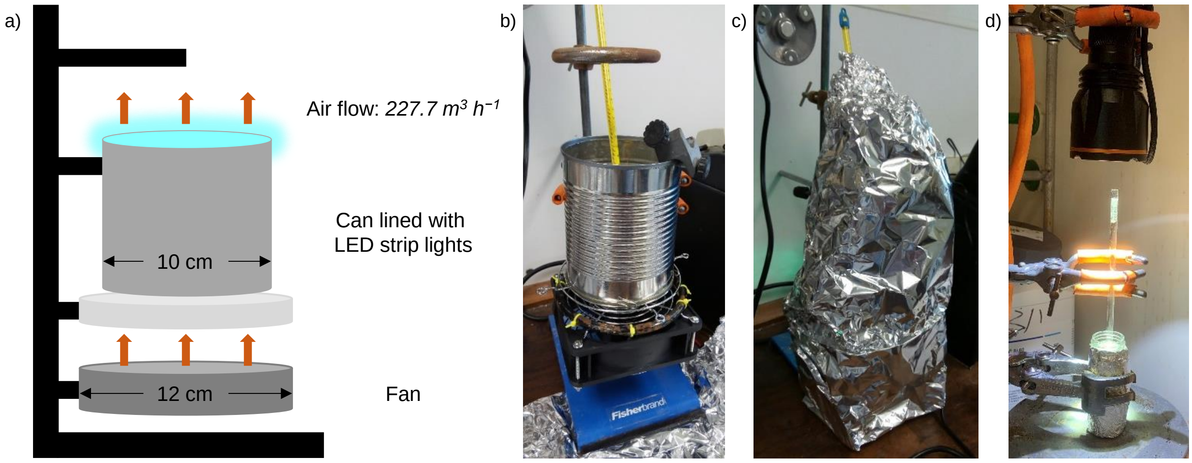

Figure S1: (a) Schematic representation of the photoreactor shown in (b). (c) Picture displaying the photoreactor in use with a LEDST60GN (green, 530 nm) LED strip. (d) Experimental set up for the Cage-Quinone Catalyzed Aza-Henry Reaction (Section 5) using a 12 W LED torch.

1.2 *In situ* reactions

The LED-NMR apparatus was constructed in-house and based on the design developed by Gshwind and co-workers.[1] A fibre-coupled LED (λ = 455 nm) powered by a BLS-SA04-US LED driver was purchased from Mightex Systems. This is connected to an Opt 20L TTL trigger box from Hi-Tech Scientific, fitted with a BNC splitter to allow simultaneous communication with the NMR console and the LED driver. The operation of an external trigger switch by the experimenter thus causes the spectrometer to begin recording measurements simultaneously to the reaction beginning to be illuminated. The fibre-optic cable is a FP1500URT purchased from Thor Labs. In order to prepare the fibre for our desired purpose the various layers of cladding (external rubber, followed by Teflon fibres, then a loose plastic sheath, then several layers of adhered polymer) were carefully stripped away from one end with a scalpel to the appropriate length and the final layer of optical coating was removed by dissolution in acetone. The end of the cable was very gently roughened with sandpaper to give uniform illumination in all directions, placed within a quartz coaxial insert and permanently attached using epoxy resin. All experiments are conducted within amberised standard 5 mm NMR tubes containing the fibre-optic cable held within the quartz insert to give a total reaction volume of 280 µL. Amberised NMR tubes and quartz coaxial inserts were both purchased from Norrell Scientific.

All LED-NMR experiments were performed on a Bruker Avance III 400 MHz spectrometer equipped with a Prodigy cryoprobe. The reactions were performed in CD_2Cl_2 and monitored by ^1H NMR spectroscopy, using a series of single scan spectra (zg30, acquisition time = 2 s) with a set delay between them. In order to ensure accurate measurement of the very early periods of the reaction, the pulse program was set up such that the first spectrum was acquired simultaneously to the irradiation of the sample beginning. To ensure that sufficient time had passed to allow the sample to fully relax between NMR excitations the delay between individual single-scan spectra was never shorter than 5 s.

2 Synthesis

C1,[2] *N*-phenyl-1,2,3,4-tetrahydroisoquinoline,[3] tetrakis(pyridyl)palladium(II) tetrakis[3,5-bis (trifluoromethyl)phenyl]borate ($Pd(py)_4{\cdot}2BArF$)[4] and tris(2,2'-bipyrazine)ruthenium(II) tetrakis[3,5-bis (trifluoromethyl)phenyl]borate ($Ru(bpz)_3(BArF)_2$)[5] were synthesized according to known literature procedures. Isoprene and 1,3-cyclohexadiene were purified by vacuum distillation.

3 Cage-Quinone Catalyzed *trans*-Anethole and Isoprene Cycloaddition Reactions

3.1 Ambient Light

3.1.1 General Procedure

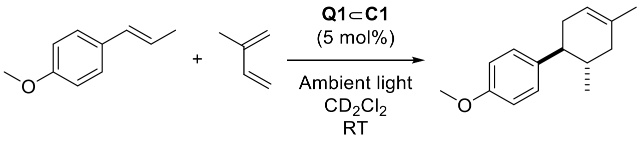

Stock solutions in CD_2Cl_2 were prepared for: 2-fluoromesitylene (internal standard, 1.20 mmol/mL), *trans*-anethole (2.40 mmol/mL), isoprene (24.0 mmol/mL) and **C1** (3.0×10^{-3} mmol/mL). A stock solution in CH_2Cl_2 was prepared for **Q1** (1.0×10^{-3} mmol/mL).

Q1 (0.09 mg, 3.0×10^{-4} mmol, 0.05 eq., 300 µL) was added to an NMR tube. The solvent was removed under a stream of N_2 and the tube was dried *in vacuo* for 1 h. To the NMR tube, CD_2Cl_2 (425 µL), internal standard (0.4 mg, 3.0×10^{-3} mmol, 0.5 eq., 25 µL), *trans*-anethole (0.9 mg, 6.0×10^{-3} mmol, 1.0 eq., 25 µL) and isoprene (4.1 mg, 6.0×10^{-2} mmol, 10.0 eq., 25 µL) were added. The reaction was initiated by addition of **C1** (1.4 mg, 3.0×10^{-4} mmol, 0.05 eq., 100 µL). The reaction was monitored by recording ^{1}H NMR spectra before and at 6 hourly intervals after the addition of **C1**. Product yields were calculated by comparing integrals of the starting material and product to that of the internal standard.

3.1.1.1 Control Reactions

To ensure all components were essential for reactivity, a variety of different control reactions were carried out. These followed the above procedure and included the following changes:

- Omitting light (amberised NMR tube).
- Omitting **Q1**.
- Omitting **C1**.

Where species were omitted, stock solution volumes were replaced with CD_2Cl_2.

3.1.2 Product Identification

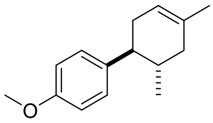

The product of each NMR scale reaction was identified (and thereby yields calculated) by comparing the spectra to previously reported ^{1}H NMR spectroscopic data.[6]

3.1.3 ^{1}H NMR spectra of ambient light catalysed [4+2] reactions

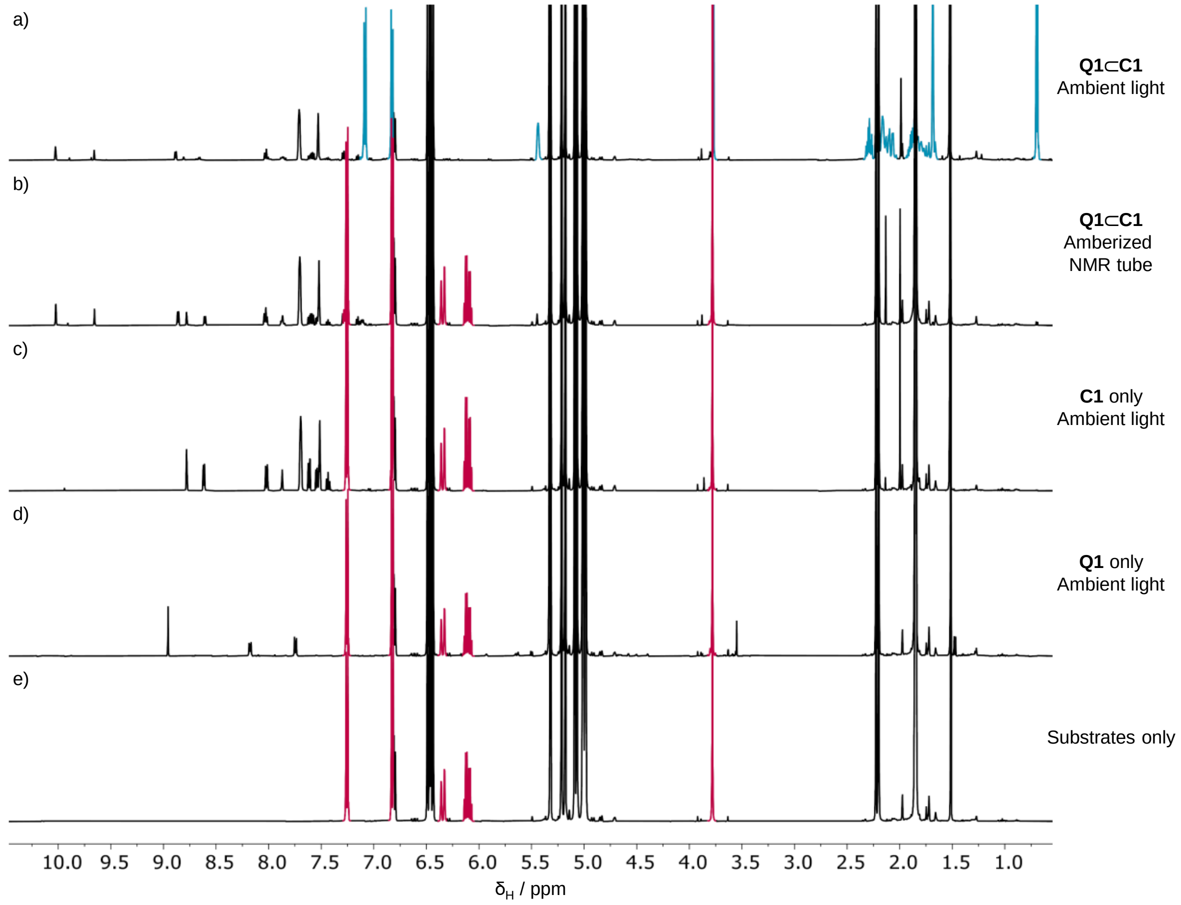

Figure S2: ^{1}H NMR spectra (500 MHz, CD_2Cl_2, 300 K) for the reaction of *trans*-anethole (10 mM) and isoprene (100 mM) in the presence of (a) **C1** (0.5 mM) and **Q1** (0.5 mM), ambient light at t = 24 h; (b) **C1** (0.5 mM) and **Q1** (0.5 mM), amberised NMR tube at t = 24 h; (c) **C1** (0.5 mM) only, ambient light at t = 24 h; (d) **Q1** (0.5 mM) only, ambient light at t = 24 h; (e) *trans*-anethole and isoprene only. *trans*-Anethole is highlighted in red, cycloadduct is highlighted in blue.

3.2 LED Irradiation

3.2.1 General Procedure

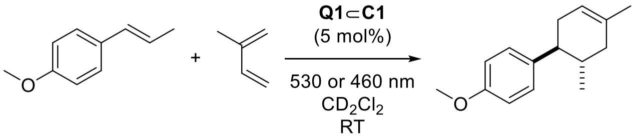

Stock solutions in CD_2Cl_2 were prepared for: 2-fluoromesitylene (internal standard, 1.20 mmol/mL), *trans*-anethole (2.40 mmol/mL) and **C1** (3.0 × 10^{-3} mmol/mL). A stock solution in CH_2Cl_2 was prepared for **Q1** (1.0 × 10^{-3} mmol/mL).

Q1 (0.09 mg, 3.0 × 10^{-4} mmol, 0.05 eq., 300 µL) was added to an NMR tube. The solvent was removed under a stream of N_2 and the tube was dried *in vacuo* for 1 h. To the NMR tube, CD_2Cl_2 (450 µL), internal standard (0.4 mg, 3.0 × 10^{-3} mmol, 0.5 eq., 25 µL), *trans*-anethole (0.9 mg, 6.0 × 10^{-3} mmol, 1.0 eq., 25 µL) and isoprene (24 µL, 16.3 mg, 0.24 mmol, 40.0 eq.) were added. The reaction was irradiated following the addition of **C1** (1.4 mg, 3.0 × 10^{-4} mmol, 0.05 eq., 100 µL). Product yields were calculated by comparing integrals of the starting material and product to that of the internal standard.

3.2.1.1 530 nm irradiation

The reaction was irradiated with 530 nm light for 5 min, followed by 10 min (15 min total), followed by 45 min (1 h total). The reaction was monitored by recording ^1H NMR spectra before the addition of **C1** and after each irradiation session.

3.2.1.1.1 ^1H NMR spectra of 530 nm light catalysed [4+2] reactions

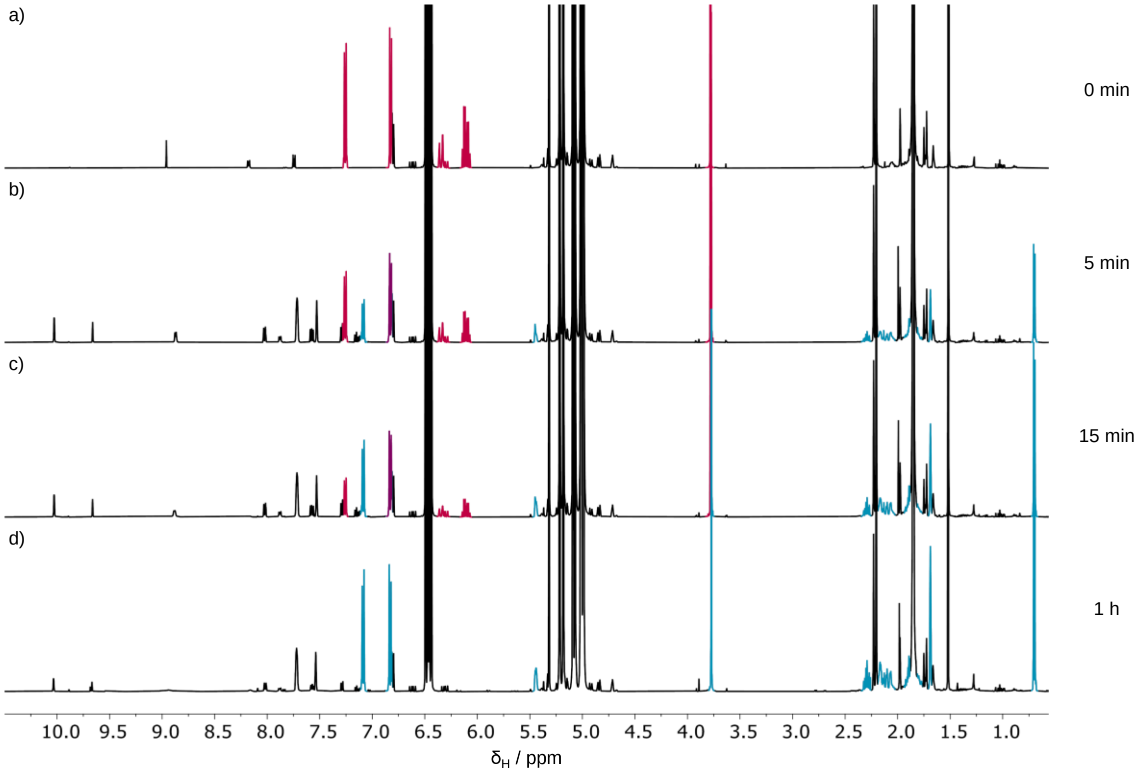

Figure S3: ^1H NMR spectra (500 MHz, CD_2Cl_2, 300 K) for the reaction of *trans*-anethole (10 mM) and isoprene (400 mM) in the presence of **C1** (0.5 mM) and **Q1** (0.5 mM) following irradiation with 530 nm light. (a) *trans*-Anethole, isoprene and **Q1**. (b) *trans*-Anethole, isoprene, **C1** and **Q1** irradiated with 530 nm light at (b) t = 5 min; (c) t = 15 min; (d) t = 1 h. *trans*-Anethole is highlighted in red, cycloadduct is highlighted in blue, overlapping *trans*-anethole and cycloadduct peak highlighted in purple.

3.2.1.2 460 nm irradiation

The reaction was irradiated with 460 nm light for 5 s, 30 s or 1 min. The reaction was monitored by recording ^{1}H NMR spectra before the addition of **C1** and after irradiation.

3.2.1.2.1 ^{1}H NMR spectra of 460 nm light catalysed [4+2] reactions

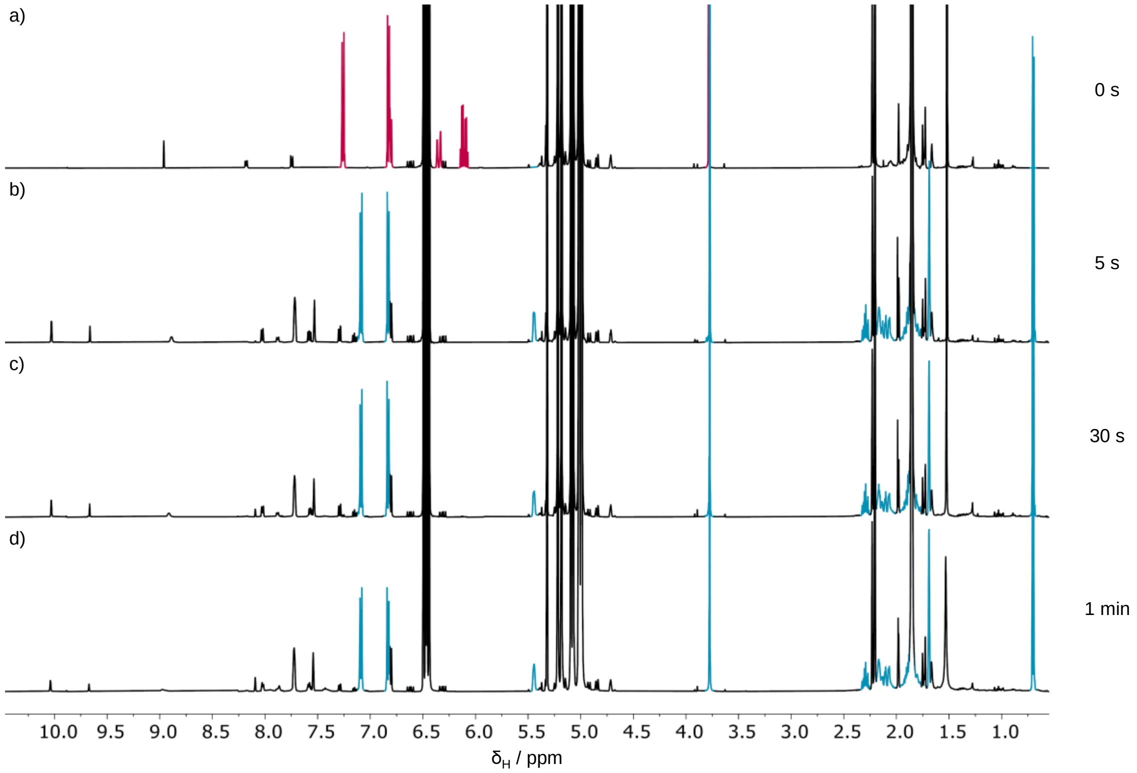

Figure S4: ^{1}H NMR spectra (500 MHz, CD_2Cl_2, 300 K) for the reaction of *trans*-anethole (10 mM) and isoprene (400 mM) in the presence of **C1** (0.5 mM) and **Q1** (0.5 mM) following irradiation with green LEDs (460 nm). (a) *trans*-Anethole, isoprene and **Q1**. (b) *trans*-Anethole, isoprene, **C1** and **Q1** irradiated with 460 nm light at (b) t = 5 s; (c) t = 30 s; (d) t = 1 min. *trans*-Anethole is highlighted in red, cycloadduct is highlighted in blue.

3.2.1.2.2 Control Reactions

To ensure all components were essential for reactivity, a variety of different control reactions were carried out. These followed the above procedure (460 nm light, t = 30 s) and included the following changes:

- Omitting **C1**.
- Omitting **C1** and replacing with $Pd(py)_4 \cdot 2BArF$.
- Omitting **C1** and replacing with 1,3-bis(pyridin-3-ylethynyl)benzene.

Where species were omitted, stock solution volumes were replaced with CD_2Cl_2. For the mono palladium species reaction, **C1** was replaced with $Pd(py)_4 \cdot 2BArF$ (1.3 mg, 6.0 × 10^{-4} mmol, 0.10 eq.). For the free ligand reaction, **C1** was replaced with 1,3-bis(pyridin-3-ylethynyl)benzene (0.3 mg, 1.2 × 10^{-3} mmol, 0.20 eq.).

3.2.1.2.2.1 ^{1}H NMR spectra of 460 nm light control [4+2] reactions

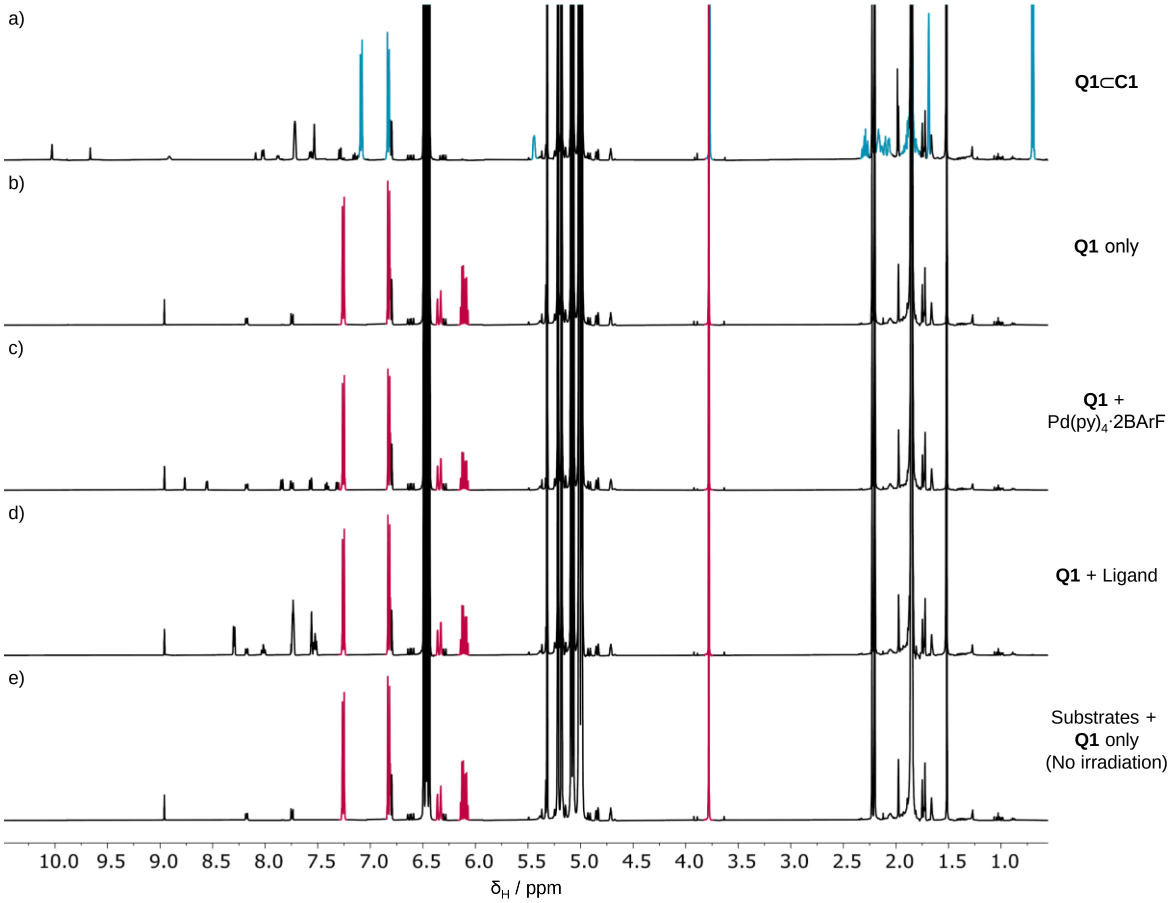

Figure S5: ^{1}H NMR spectra (500 MHz, CD_2Cl_2, 300 K) for the reaction of *trans*-anethole (10 mM) and isoprene (400 mM) after irradiation with 460 nm light at t = 30 s in the presence of (a) **C1** (0.5 mM) and **Q1** (0.5 mM); (b) **Q1** (0.5 mM) only; (c) $Pd(py)_4{\cdot}2BArF$ (1 mM) and **Q1** (0.5 mM); (d) 1,3-bis(pyridin-3-ylethynyl)benzene (2 mM) and **Q1** (0.5 mM). (e) *trans*-anethole, isoprene and **Q1** only, no irradiation. *trans*-Anethole is highlighted in red, cycloadduct is highlighted in blue.

3.3 Catalyst Loading Experiments

3.3.1 General Procedure

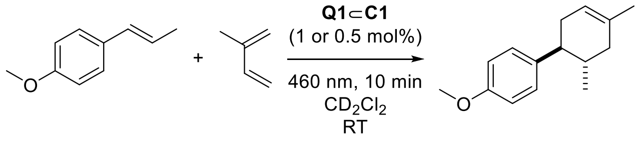

A stock solution of **Q1** (1.2 mg, 4 x 10^{-3} mmol) in CH_2Cl_2 (3.0 mL) was prepared. *trans*-Anethole (10 mg, 7.0 × 10^{-2} mmol, 1.0 eq.), isoprene (270 µL, 184 mg, 2.7 mmol, 40 eq.) and **Q1** (0.1 or 0.2 mg, 3.4 or 6.7 × 10^{-4} mmol, 125 or 250 µL, for 0.5 or 1 mol%, respectively) were placed into a microwave vial containing CH_2Cl_2 (3.6 or 3.5 mL, for 0.5 or 1 mol%, respectively). In the absence of light, **C1** (1.6 or 3.2 mg, 3.4 × 10^{-4} or 6.7 x10^{-4} mmol, for 0.5 or 1 mol%, respectively) was added and the microwave vial was sealed. An aliquot (400 µL) was immediately taken and added to an NMR tube containing CD_2Cl_2 (200 µL), which was kept in the dark to avoid photoreaction. The solution was irradiated with 460 nm light for 10 min. Another aliquot (400 µL) was taken and added to an NMR tube containing CD_2Cl_2 (200 µL). Product yields were calculated by comparing integrals of the starting material and product to that of the internal standard.

3.3.2 ^{1}H NMR spectra of catalyst loading [4+2] reactions

3.3.2.1 1 mol% **Q1⊂C1**

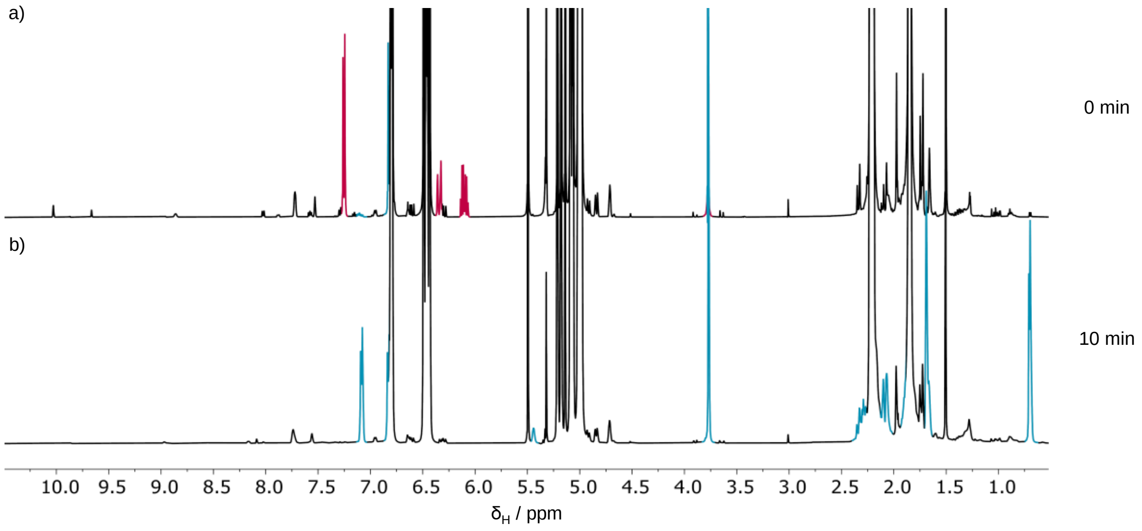

Figure S6: ^{1}H NMR spectra (500 MHz, CD_2Cl_2/CH_2Cl_2, 300 K, with solvent suppression) for the reaction of *trans*-anethole (17 mM) and isoprene (675 mM) in the presence of **C1** (0.2 mM) and **Q1** (0.2 mM) (a) at t = 0 min; (b) after irradiation with 460 nm light, t = 10 min. *trans*-Anethole is highlighted in red, cycloadduct is highlighted in blue.

3.3.2.2 0.5 mol% **Q1⊂C1**

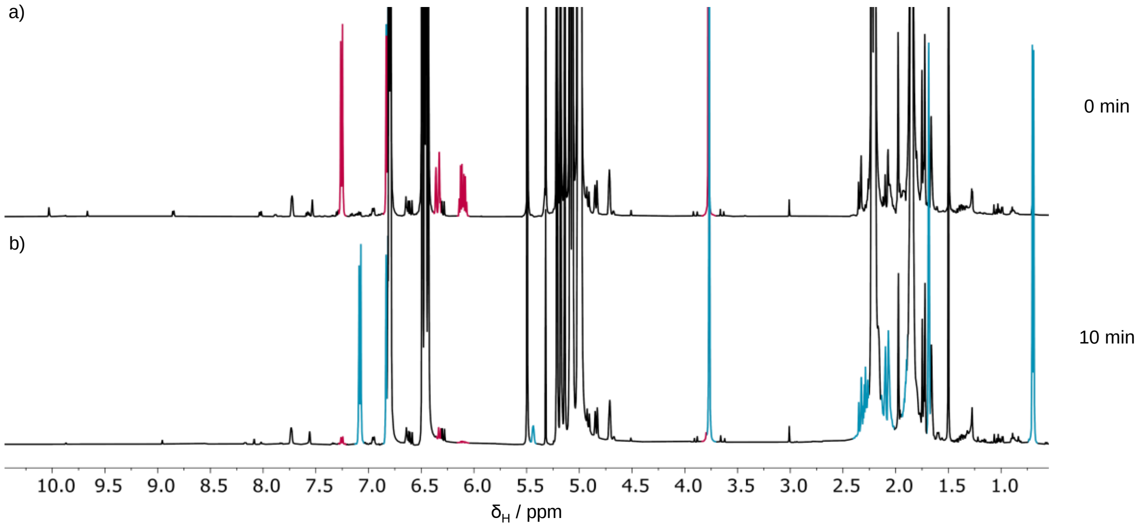

Figure S7: ^{1}H NMR spectra (500 MHz, CD_2Cl_2/CH_2Cl_2, 300 K, with solvent suppression) for the reaction of *trans*-anethole (17 mM) and isoprene (675 mM) in the presence of **C1** (0.1 mM) and **Q1** (0.1 mM) (a) at t = 0 min; (b) after irradiation with 460 nm light, t = 10 min. *trans*-Anethole is highlighted in red, cycloadduct is highlighted in blue.

3.4 Preparative Scale Reaction

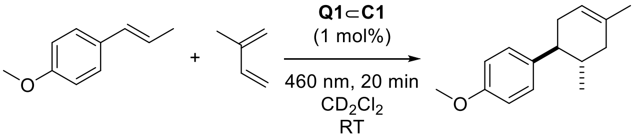

A stock solution of **Q1** (4.0 mg, 0.013 mmol) in CH_2Cl_2 (4.0 mL) was prepared. *trans*-Anethole (50 mg, 0.34 mmol, 1.0 eq.), isoprene (1.35 mL, 920 mg, 13.5 mmol, 40 eq.) and **Q1** (1.0 mg, 3.4×10^{-3} mmol, 0.01 eq., 1 mL) were placed into a microwave vial containing CH_2Cl_2 (3.6 mL). **C1** (16.2 mg, 3.4×10^{-3} mmol, 0.01 eq.) was added and the microwave vial was sealed. The solution was irradiated with blue LED light (460 nm) for 20 min at room temperature under a constant stream of compressed air. The solvent was removed *in vacuo*. The residues were dissolved in hexanes, filtered through a 17 mm PTFE 0.45 µm syringe filter and the solvent removed *in vacuo* to give the cycloadduct as a colourless oil (66 mg, 0.3 mmol, 90%). **^{1}H NMR (500 MHz, $CDCl_3$) δ_H:** 7.10 – 7.06 (m, 2H), 6.86 – 6.82 (m, 2H), 5.47 – 5.42 (m, 1H), 3.79 (s, 3H), 2.30 (dt, J = 10.7, 5.6 Hz, 1H), 2.25 – 2.05 (m, 3H), 1.94 – 1.75 (m, 2H), 1.69 (s, 3H), 0.71 (d, J = 6.4 Hz, 3H).

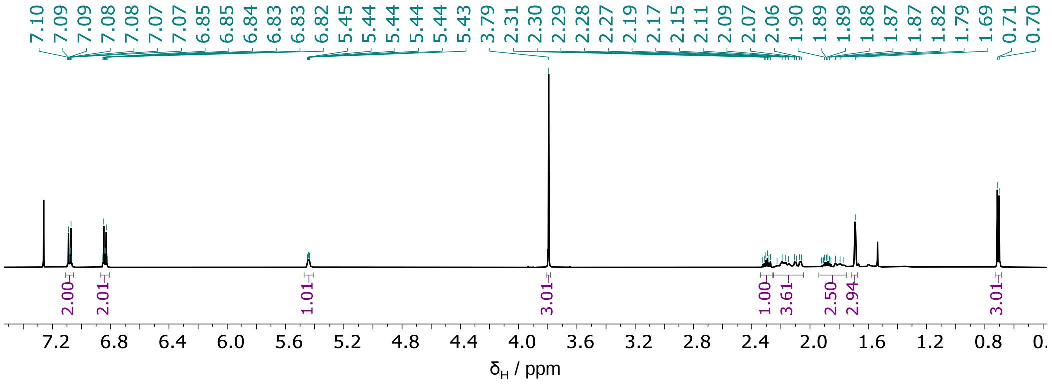

Figure S8: ^{1}H NMR spectrum (500 MHz, $CDCl_3$, 300 K) for the cycloadduct isolated from the reaction of *trans*-anethole and isoprene in the presence of **C1** and **Q1** and 460 nm light.

3.5 Quinone Scope

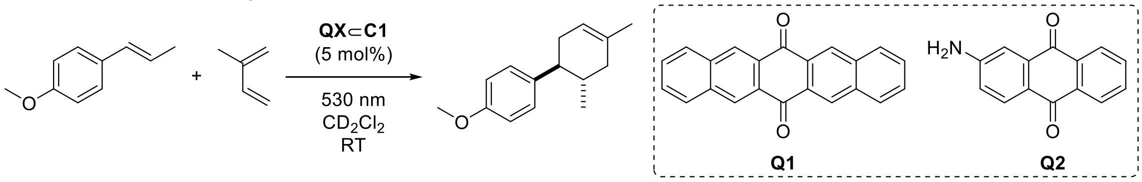

Stock solutions in CD_2Cl_2 were prepared for: 2-fluoromesitylene (internal standard, 1.20 mmol/mL), *trans*-anethole (2.40 mmol/mL) and **C1** (3.0×10^{-3} mmol/mL). A stock solution in CH_2Cl_2 was prepared for **Q1** (1.0×10^{-3} mmol/mL) and **Q2** (1.0×10^{-3} mmol/mL).

Q1 (0.09 mg, 3.0×10^{-4} mmol, 0.05 eq., 300 µL) or **Q2** (0.07 mg, 3.0×10^{-4} mmol, 0.05 eq., 300 µL) was added to an NMR tube. The solvent was removed under a stream of N_2 and the tube was dried *in vacuo* for 1 h. To the NMR tube, CD_2Cl_2 (450 µL), internal standard (0.4 mg, 3.0×10^{-3} mmol, 0.5 eq., 25 µL), *trans*-anethole (0.9 mg, 6.0×10^{-3} mmol, 1.0 eq., 25 µL) and isoprene (24 µL, 16.3 mg, 0.24 mmol, 40.0 eq.) were added. The reaction was initiated with green LED (530 nm) irradiation following the addition of **C1** (1.4 mg, 3.0×10^{-4} mmol, 0.05 eq., 100 µL). The reactions were irradiated for 5 min, followed by 10 min (15 min total), followed by 45 min (1 h total). The reactions were monitored by recording ^{1}H NMR spectra before the addition of **C1** and after each irradiation session. Product yields were calculated by comparing integrals of the starting material and product to that of the internal standard.

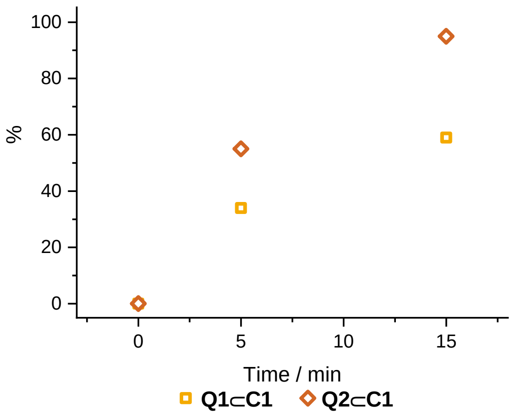

Figure S9: Percent formation of cycloadduct over time for the reaction of *trans*-anethole (10 mM) and isoprene (400 mM) irradiated with 460 nm light in the presence of **C1** (0.5 mM) and (i) **Q1** (0.5 mM, yellow squares); (ii) **Q2** (0.5 mM, orange diamonds).

3.5.1 ^{1}H NMR spectra of [4+2] quinone scope reactions

3.5.1.1 Q1

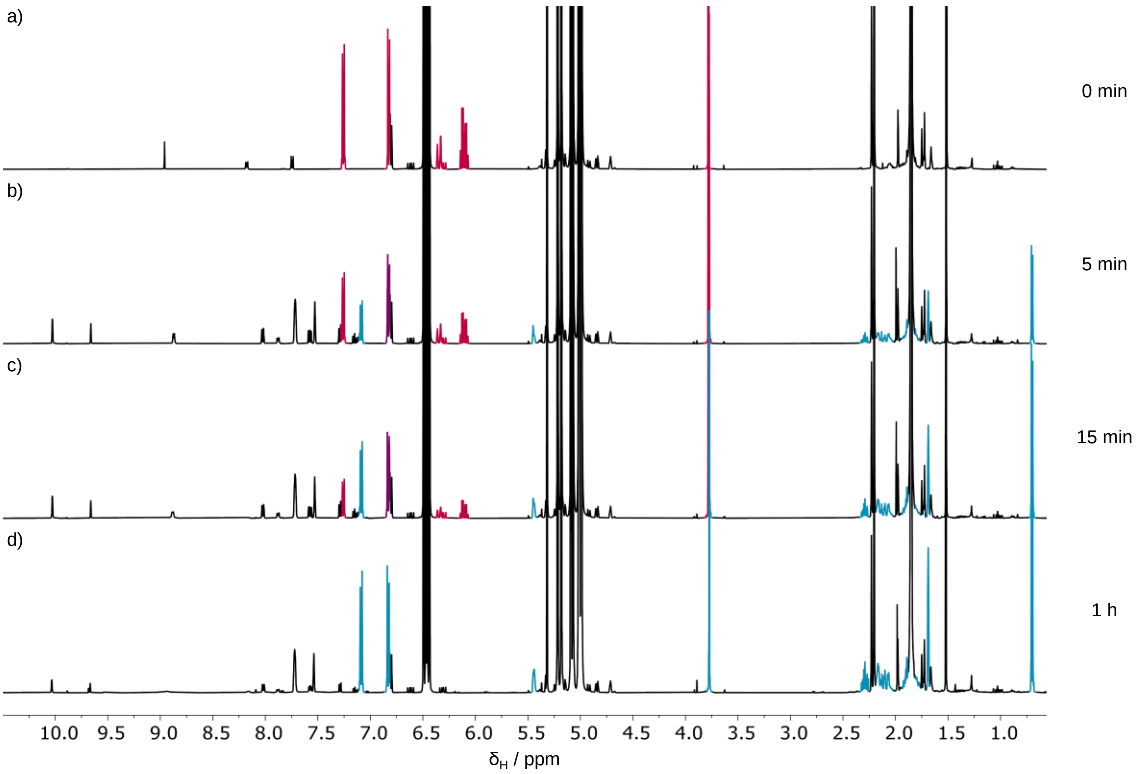

Figure S10: ^{1}H NMR spectra (500 MHz, CD_2Cl_2, 300 K) for the reaction of *trans*-anethole (10 mM) and isoprene (400 mM) in the presence of **C1** (0.5 mM) and **Q1** (0.5 mM) following irradiation with 530 nm light. (a) *trans*-Anethole, isoprene and **Q1**. (b) *trans*-Anethole, isoprene, **C1** and **Q1** irradiated with 530 nm light at (b) t = 5 min; (c) t = 15 min; (d) t = 1 h. *trans*-Anethole is highlighted in red, product is highlighted in blue, overlapping *trans*-anethole and cycloadduct peak highlighted in purple.

3.5.1.2 **Q2**

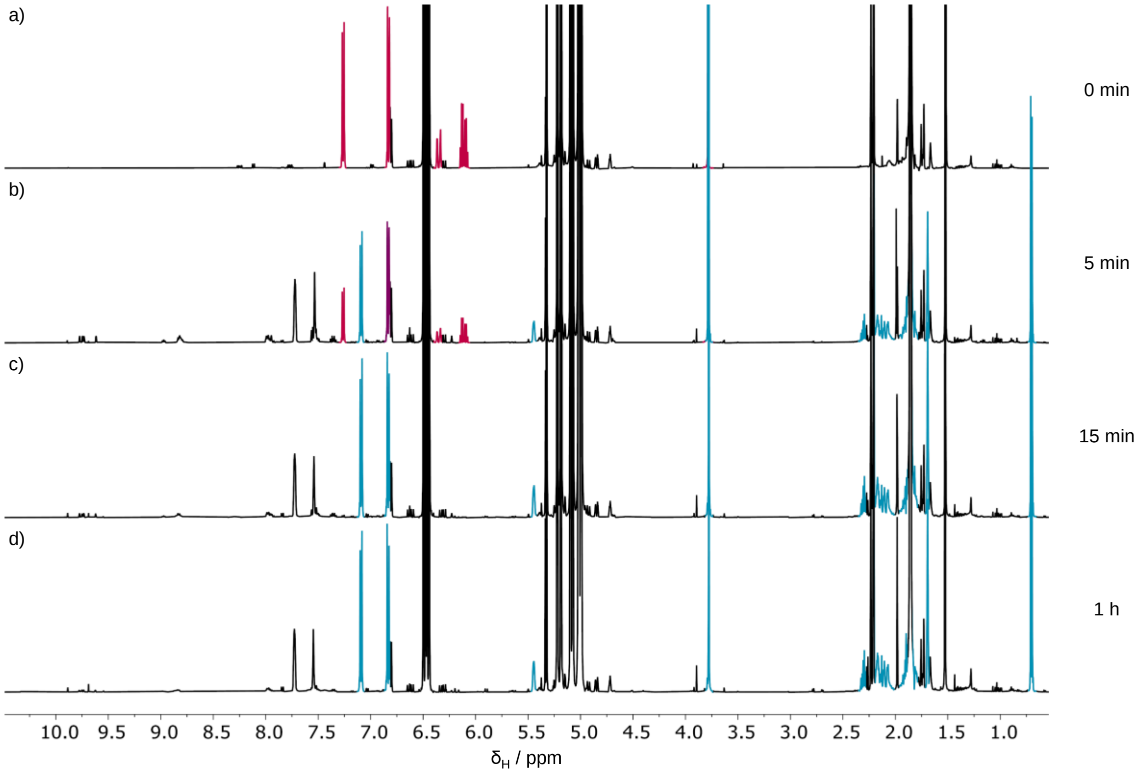

Figure S11: ^{1}H NMR spectra (500 MHz, CD_2Cl_2, 300 K) for the reaction of *trans*-anethole (10 mM) and isoprene (400 mM) in the presence of **C1** (0.5 mM) and **Q2** (0.5 mM) following irradiation with 530 nm light. (a) *trans*-Anethole, isoprene and **Q1**. (b) *trans*-Anethole, isoprene, **C1** and **Q2** irradiated with 530 nm light at (b) t = 5 min; (c) t = 15 min; (d) t = 1 h. *trans*-Anethole is highlighted in red, cycloadduct is highlighted in blue, overlapping *trans*-anethole and cycloadduct peak highlighted in purple.

3.6 *In situ* Illumination NMR Spectroscopy Kinetics Experiments

3.6.1 General Procedure

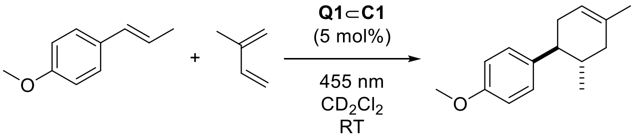

Stock solutions in CD_2Cl_2 were prepared for: 2-fluoromesitylene (internal standard, 1.20 mmol/mL), *trans*-anethole (2.40 mmol/mL) and **C1** (3.0×10^{-3} mmol/mL). A stock solution in CH_2Cl_2 was prepared for **Q1** (1.0×10^{-3} mmol/mL).

3.6.1.1 5 mol% Catalyst Loadings

Q1 (0.09 mg, 3.0×10^{-4} mmol, 0.05 eq., 300 µL) was added to an NMR tube. The solvent was removed under a stream of N_2 and the tube was dried *in vacuo* for 1 h. To the NMR tube, CD_2Cl_2 (450 µL), internal standard (0.4 mg, 3.0×10^{-3} mmol, 0.5 eq., 25 µL), *trans*-anethole (0.9 mg, 6.0×10^{-3} mmol, 1.0 eq., 25 µL), isoprene (24 µL, 16.3 mg, 0.24 mmol, 40.0 eq.) and **C1** (1.4 mg, 3.0×10^{-4} mmol, 0.05 eq., 100 µL) were added. In order that each interleaved data set contained identical solutions, several of these solutions were combined in an amberised HPLC vial and fully homogenised before use.

3.6.1.2 1 mol% Catalyst Loadings

Q1 (0.02 mg, 6.0×10^{-5} mmol, 0.01 eq., 60 µL) was added to an NMR tube. The solvent was removed under a stream of N_2 and the tube was dried *in vacuo* for 1 h. To the NMR tube, CD_2Cl_2 (530 µL), internal standard (0.4 mg, 3.0×10^{-3} mmol, 0.5 eq., 25 µL), *trans*-anethole (0.9 mg, 6.0×10^{-3} mmol, 1.0 eq., 25 µL), isoprene (24 µL, 16.3 mg, 0.24 mmol, 40.0 eq.) and **C1** (0.3 mg, 6.0×10^{-5} mmol, 0.01 eq., 20 µL) were added. In order that each light intensity data set contained identical solutions, several of these solutions were combined in an amberised HPLC vial and fully homogenized before use.

3.6.1.3 Lower Catalyst Loadings

The reactions with lower catalyst loadings (0.75, 0.5 and 0.25 mol%) were prepared as above, reducing the volumes of **Q1** and **C1** used as necessary, replacing the reduced volume of **C1** stock with CD_2Cl_2 to ensure constant overall volume.

For each NMR reaction, the solution (280 µL) was added to an amberised NMR tube, into which the coaxial insert containing the fibre-optic cable was inserted and the tube was sealed. This was lowered into the spectrometer probe and the sample was locked, tuned/matched and shimmed. An initial reference spectrum was taken prior to illumination of the sample.

Initial experimentation using 5 mol% **Q1⊂C1** loading and the maximum possible LED intensity ('100% power') revealed that the reaction was too fast to conveniently monitor, and so it was slowed down by dropping the catalyst loading to 1 mol% and standard LED intensity to 5% of the maximum. Between each reaction the quartz/fibre optic insert was thoroughly cleaned by rinsing with acetone and wiping with a paper towel.

3.6.2 Orders in light intensity and catalyst loading

Light intensity studies were conducted by varying the LED intensity and catalyst loading studies were conducted by varying the catalyst concentration of the solution. Initial rates were taken by approximating the first 40 s (5 data points for catalyst loading studies) or first 30 s (4 data points for light intensity studies) of the reactions as a straight line.

3.6.2.1 Catalyst loading

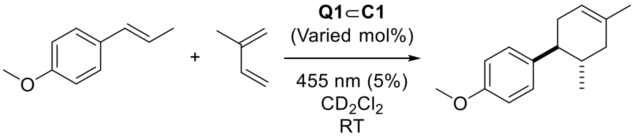

a) b)

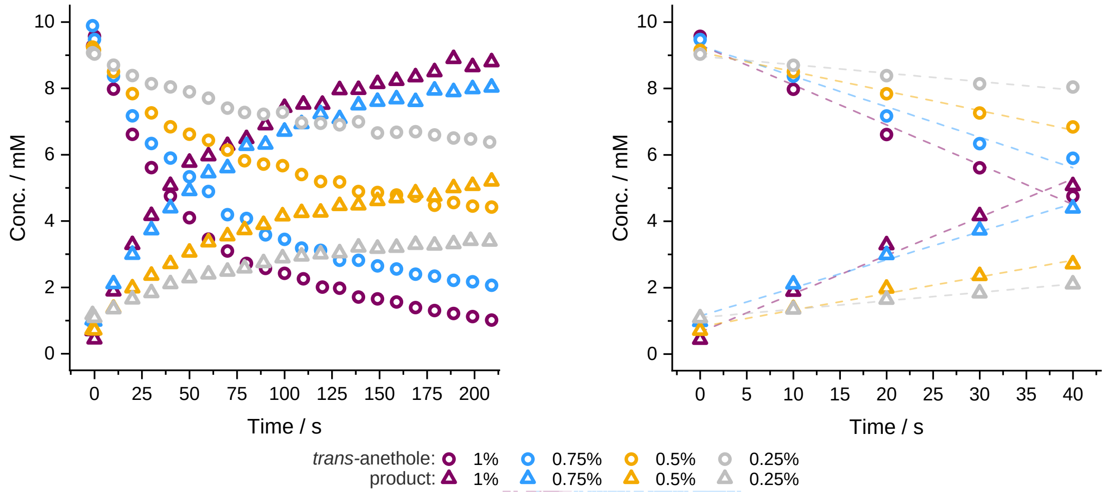

c)

Catalyst loading/ %	Initial rate / mM s^{-1}	
	trans-Anethole	Product
1	0.1200	0.1154
0.75	0.0919	0.0846
0.5	0.0586	0.0501
0.25	0.0254	0.253

d)

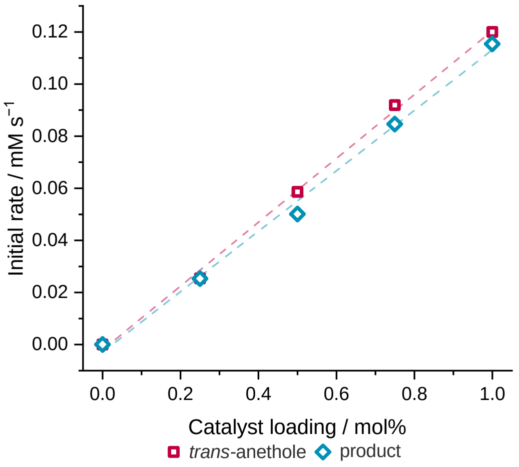

Figure S12: (a) Concentration vs time plots of consumption of *trans*-anethole (circles) and production of cycloadduct (triangles) using various mol% **Q1⊂C1** in CD_2Cl_2 with 5% LED intensity: (i) 1 mol% **Q1⊂C1** (purple circles and triangles); (ii) 0.75 mol% **Q1⊂C1** (blue circles and triangles); (iii) 0.5 mol% **Q1⊂C1** (yellow circles and triangles); (iv) 0.25 mol% **Q1⊂C1** (grey circles and triangles). (b) Linear fittings of initial points in graph (a) to yield initial rates (mM s^{-1}). (c) Initial rates obtained from the linear fittings of (b). (d) Initial rate of product formation vs catalyst loading plot shows a linear relationship.

3.6.2.2 Light intensity

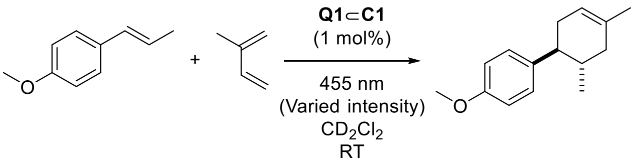

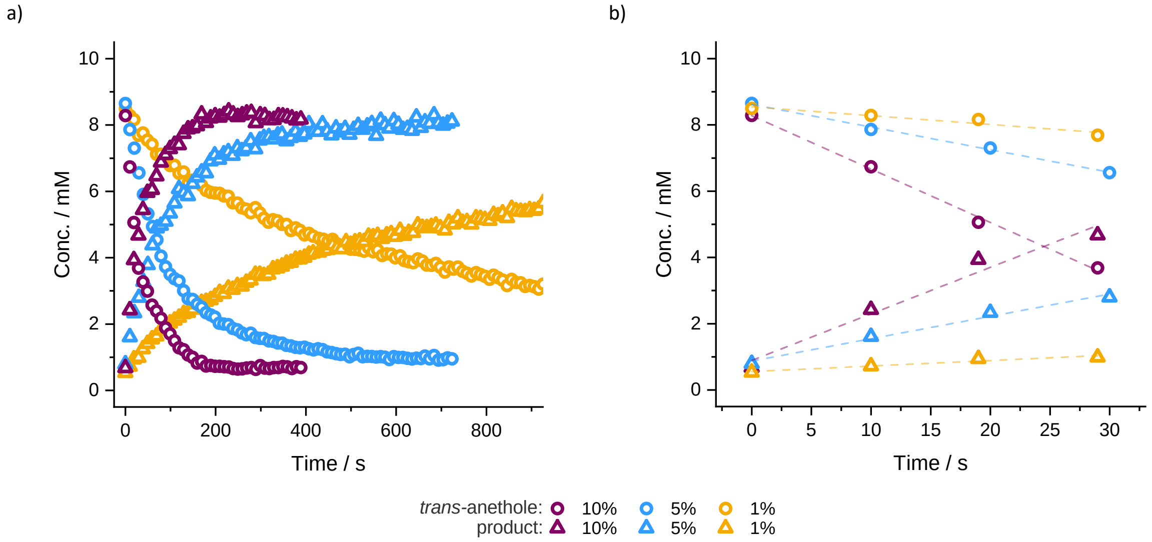

c)

Light Intensity / %	Initial rate / mM s^{-1}	
	***trans*-Anethole**	**Product**
10	0.1612	0.1405
5	0.0683	0.0674
1	0.0214	0.1405

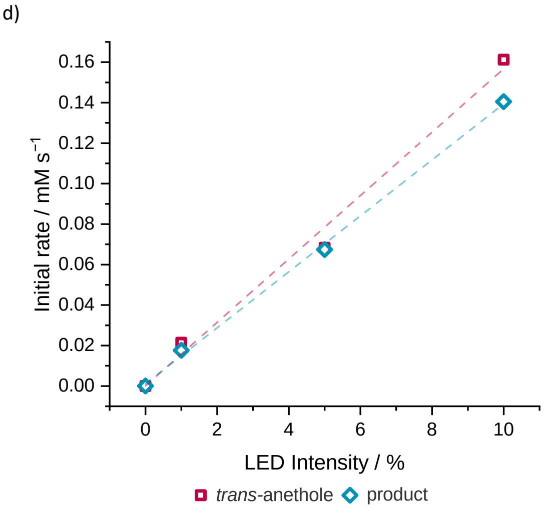

Figure S13: (a) Concentration vs time plots of consumption of *trans*-anethole (circles) and production of cycloadduct (triangles) using 5 mol% **Q1⊂C1** in CD_2Cl_2 using different LED intensities: (i) 10% LED intensity (purple circles and triangles); (ii) 5% LED intensity (blue circles and triangles); (iii) 1% LED intensity (yellow circles and triangles). (b) Linear fittings of initial points in graph (a) to yield initial rates (mM s^{-1}). (c) Initial rates obtained from the linear fittings of (b). (d) Initial rate of product formation vs LED intensity plot shows a linear relationship.

3.6.3 On/off studies

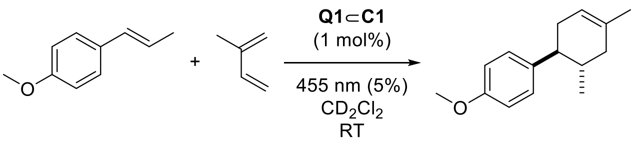

Using the programming functions of the LED driver, the reaction (with 1 mol% catalyst) was illuminated at 455 nm and 5% intensity for 60 s followed by 300 s of darkness. This process was repeated twice, and the reaction was then illuminated for a further 120 s prior to the light being turned off, although the reaction was monitored for a further 1000 s (as shown in Figure S15).

3.6.3.1 ^{1}H NMR spectra of in situ (on/off) irradiated [4+2] cycloadditions

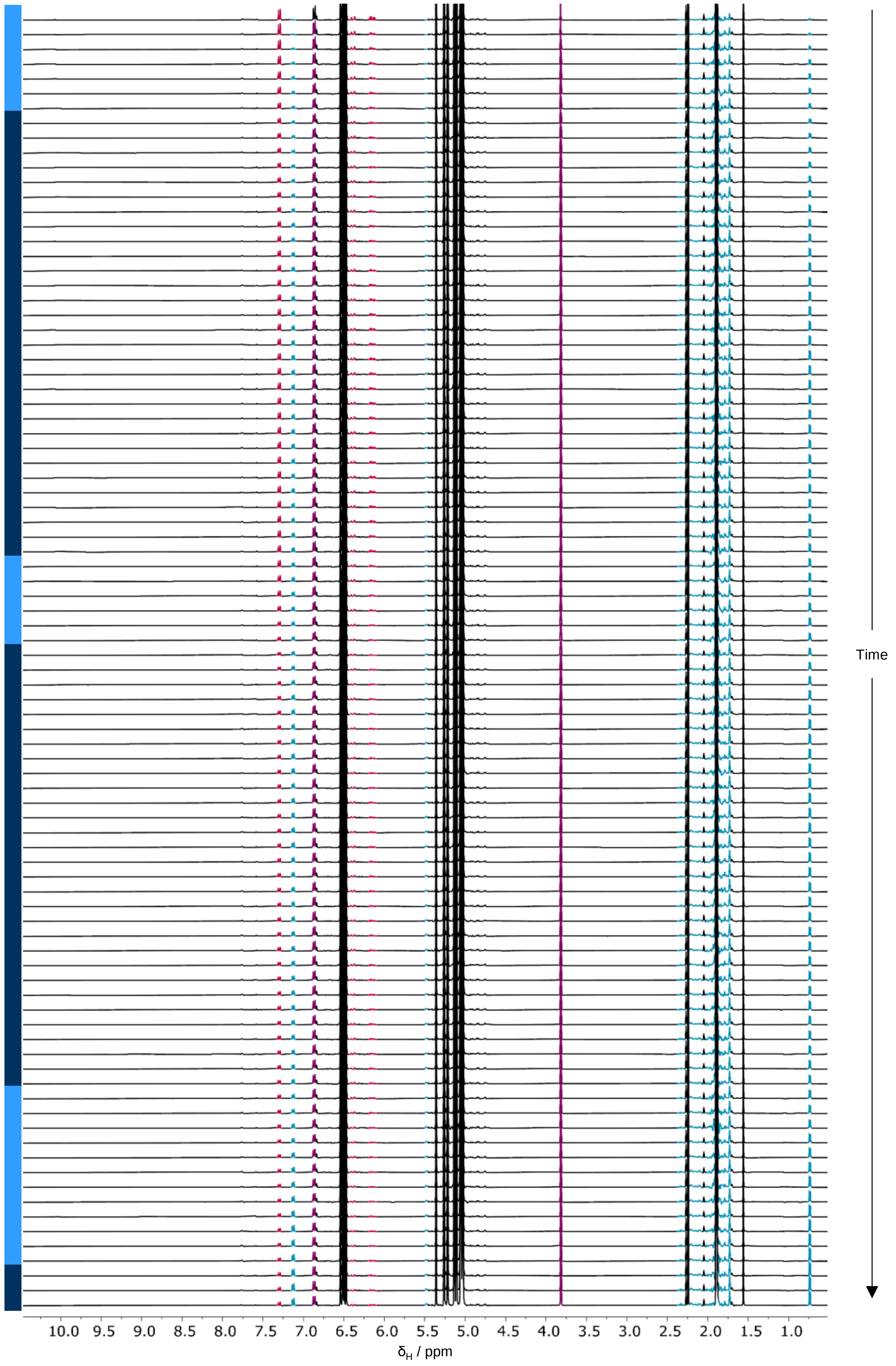

Figure S14: ^{1}H NMR spectra (400 MHz, CD_2Cl_2, 300 K) for the reaction of *trans*-anethole (10 mM) and isoprene (400 mM) in the presence of **C1** (0.1 mM) and **Q1** (0.1 mM) irradiated *in situ* with 455 nm light (5% power). Periods of irradiation indicated by the blue bar, periods of darkness indicated by the navy bar. *trans*-Anethole is highlighted in red, product is highlighted in blue, overlapping *trans*-anethole and cycloadduct peaks highlighted in purple.

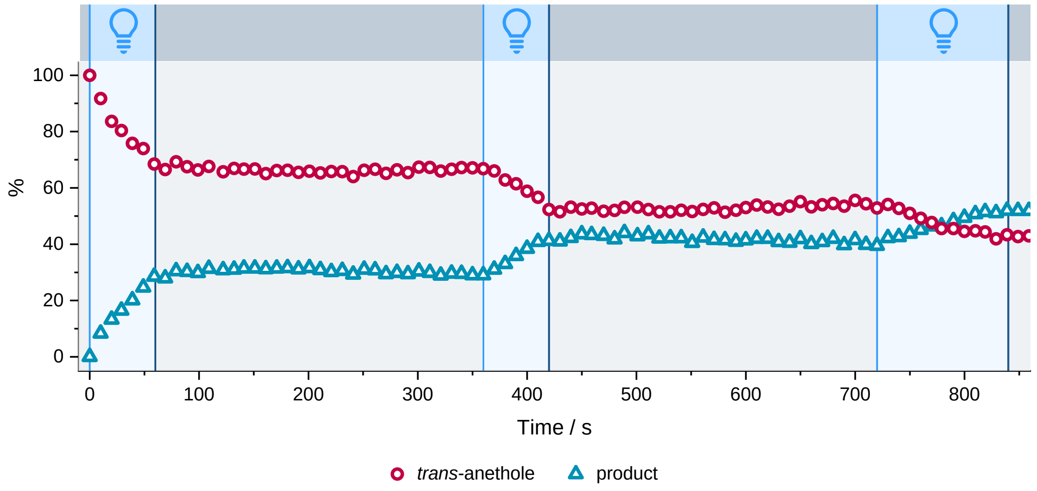

Figure S15: Light-on-light-off catalysis using 1 mol% **Q1⊂C1** and 5% LED power (455 nm) showing "stepped" profile in consumption of *trans*-anethole (red circles) and formation of product (blue triangles).

3.6.4 Measurement of the reaction kinetics at standard catalyst loadings and light intensity

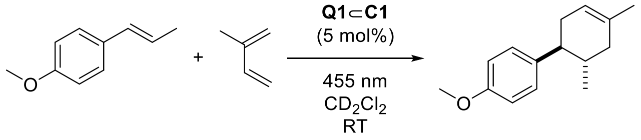

Following initial measurements that showed that the standard reaction was over within at least 10 s under LED-NMR conditions at maximum light intensity, an interleaving strategy was used to measure the form of the reaction kinetics under these conditions. A large batch of identical reaction solution at 5 mol% catalyst loading was prepared, as discussed above. A series of single-scan NMR measurements were then made, using different delays (0–5 s) between the initiation of illumination and measurement of the first data point in each fresh 280 µL sample. Each of these kinetic traces was then plotted on the same axis to give a full kinetic picture.

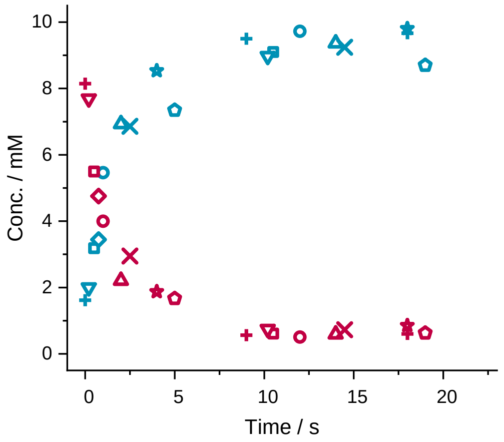

Figure S16: Concentration vs time plot of consumption of *trans*-anethole (red symbols) and production of cycloadduct (blue symbols) using 5 mol% **Q1⊂C1** in CD_2Cl_2 irradiated with 455 nm light (100%). Different data sets are shown using different symbols.

3.6.4.1 Interleaved ^{1}H NMR spectra of *in situ* irradiated [4+2] cycloadditions

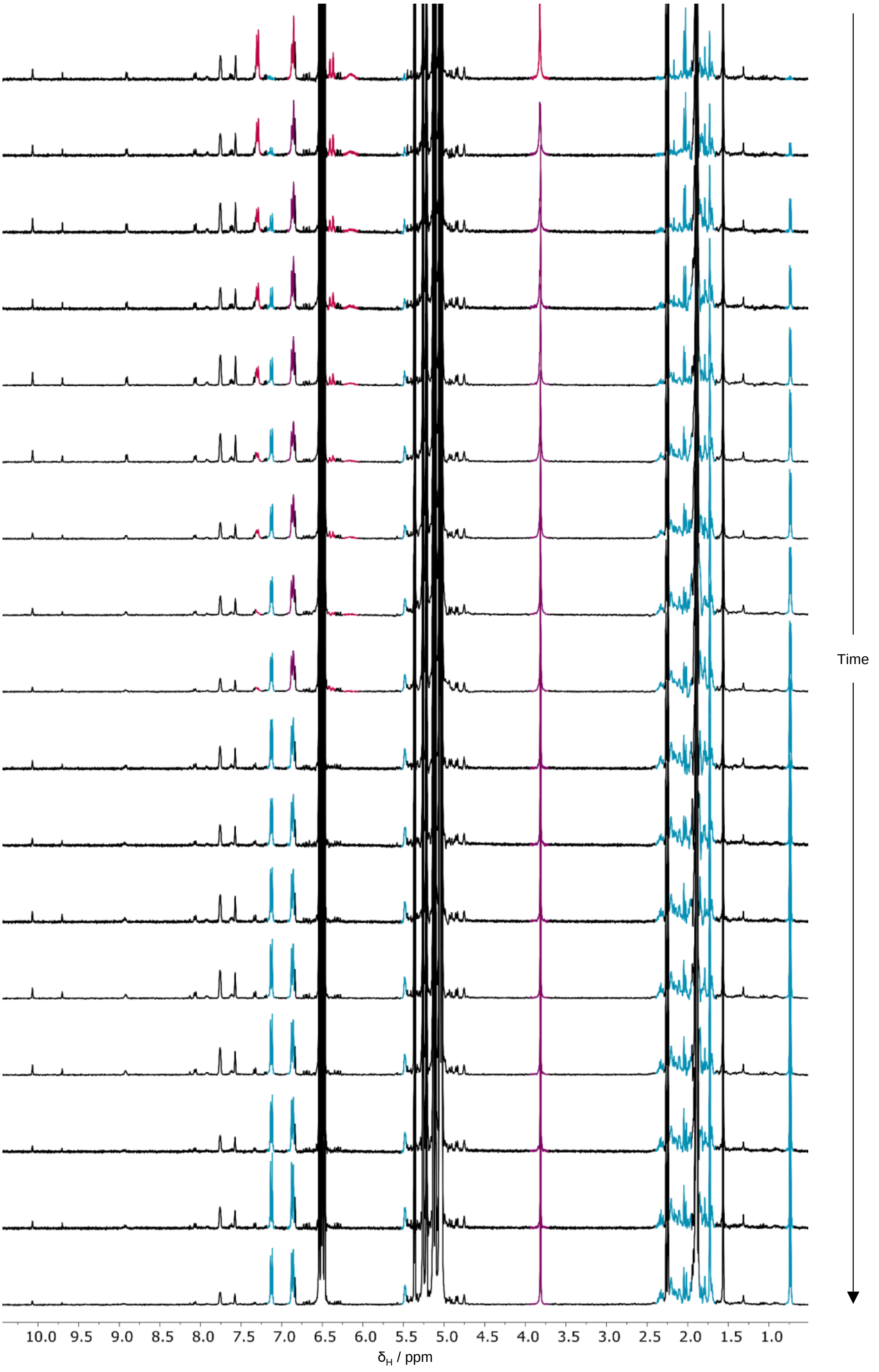

Figure S17: Interleaved ^{1}H NMR spectra (400 MHz, CD_2Cl_2, 300 K) for the reaction of *trans*-anethole (10 mM) and isoprene (400 mM) in the presence of **C1** (0.5 mM) and **Q1** (0.5 mM) irradiated *in situ* with 455 nm light (100% power) generated from 9 data sets. *trans*-Anethole is highlighted in red, product is highlighted in blue, overlapping *trans*-anethole and cycloadduct peaks highlighted in purple.

3.6.5 Use of different batches of **C1**

Two reactions were prepared identically, with the exception that each contained 1 mol% of **C1** synthesised in two distinct batches. They were both run under the same conditions with *in situ* monitoring and the resulting overlaid kinetic profiles can be seen below:

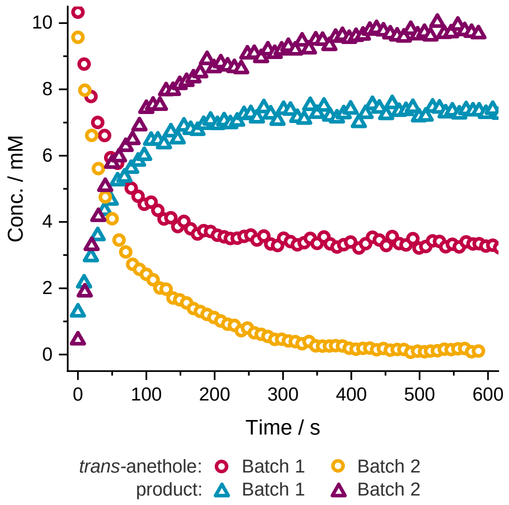

Figure S18: Overlaid kinetic plots of two identical reactions each using 1 mol% of a different batch of **C1** of the conversion of *trans*-anethole (circles) to product (triangles).

3.6.6 Comparison to existing photocatalyst Ru(bpz)3(BArF)2

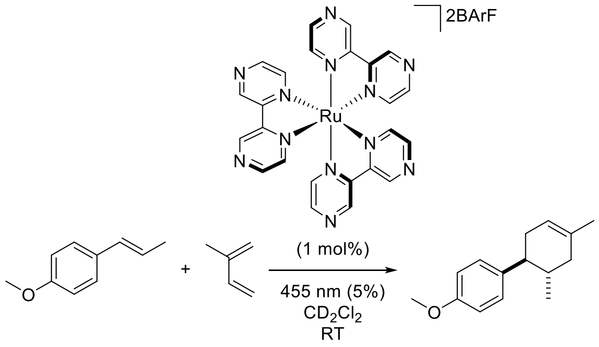

A solution consisting of 1 mol% (0.1 mM) Ru(bpz)3(BArF)2 was also prepared from stock solutions following a similar general procedure to that discussed previously, and the kinetics of this reaction under irradiation at 5% light intensity was measured using two distinct batches of Ru(bpz)3(BArF)2 under otherwise identical conditions.

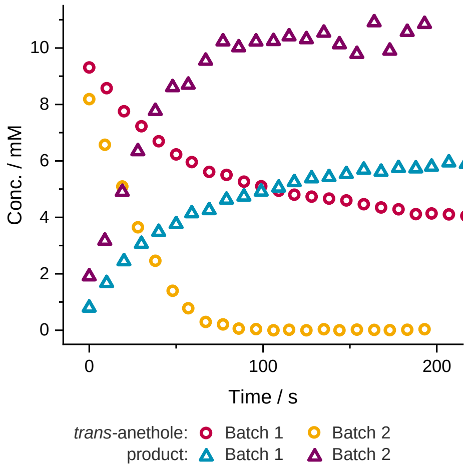

Figure S19: Overlaid kinetic plots of two identical reactions each using 1 mol% of a different batch of Ru(bpz)3(BArF)2 of the conversion of *trans*-anethole (circles) to product (triangles).

3.6.7 Addition of NaBArF to the reactions

A 280 µL aliquot was taken from a fully-assembled reaction solution. To the remainder of the solution was added sufficient NaBArF to saturate the solution. A 280 µL aliquot of this solution was placed in an amberised NMR tube and the reaction was monitored by LED-NMR. Comparison between the NaBArF-saturated solution and the solution without additional NaBArF was conducted using both 1 mol% **Q1⊂C1** and 1 mol% Ru(bpz)$_3$BArF$_2$.

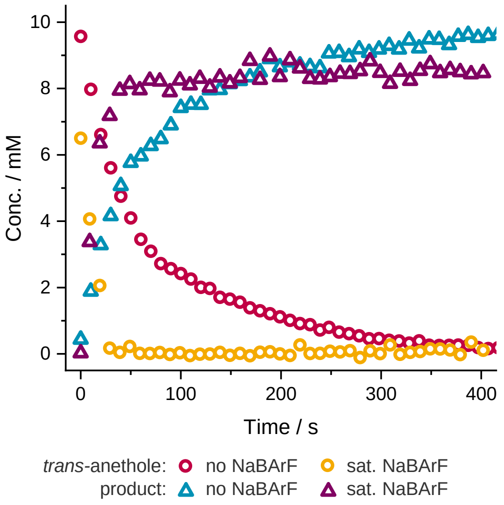

Figure S20: Overlaid kinetic plots of two otherwise-identical reactions each using 1 mol% **Q1⊂C1**, where one solution is saturated with NaBArF, of the conversion of *trans*-anethole (circles) to product (triangles).

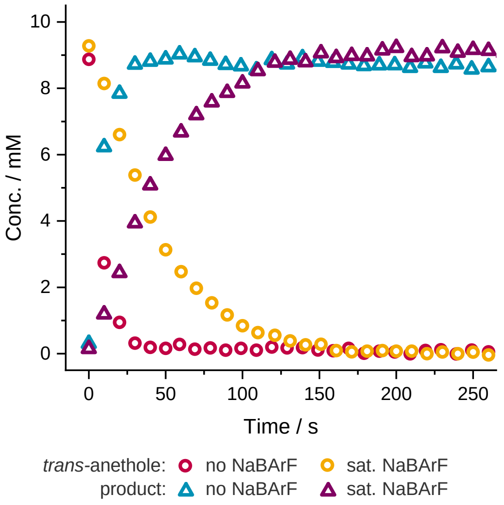

Figure S21: Overlaid kinetic plots of two otherwise-identical reactions each using 1 mol% Ru(bpz)$_3$(BArF)$_2$, where one solution is saturated with NaBArF, of the conversion of *trans*-anethole (circles) to product (triangles).

3.6.8 Reaction using only **Q1** and NaBArF

A reaction solution was prepared containing 1 mol% **Q1**. This solution was saturated with NaBArF and the resulting reactivity was monitored by LED-NMR illumination at 455 nm and 5% LED intensity. This was compared to an identical solution with no addition of NaBArF, where no reactivity was observed.

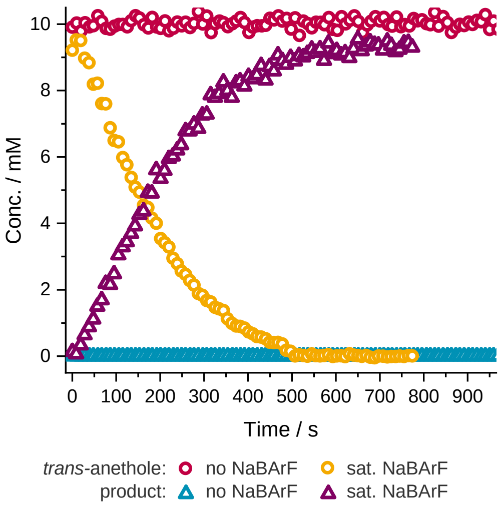

Figure S22: Overlaid kinetic plots of two otherwise-identical reactions each using 5 mol% **Q1** and no additional photocatalyst, where one solution is saturated with NaBArF, of the conversion of *trans*-anethole (circles) to product (triangles).

Using 5 mol% of NaBArF with no photocatalyst or **Q1** yielded no reactivity. Saturating a solution of only **1** and **2** in CD_2Cl_2 with NaBArF yielded trace amounts of product after 15 minutes of irradiation at 455 nm (see Figure S23).

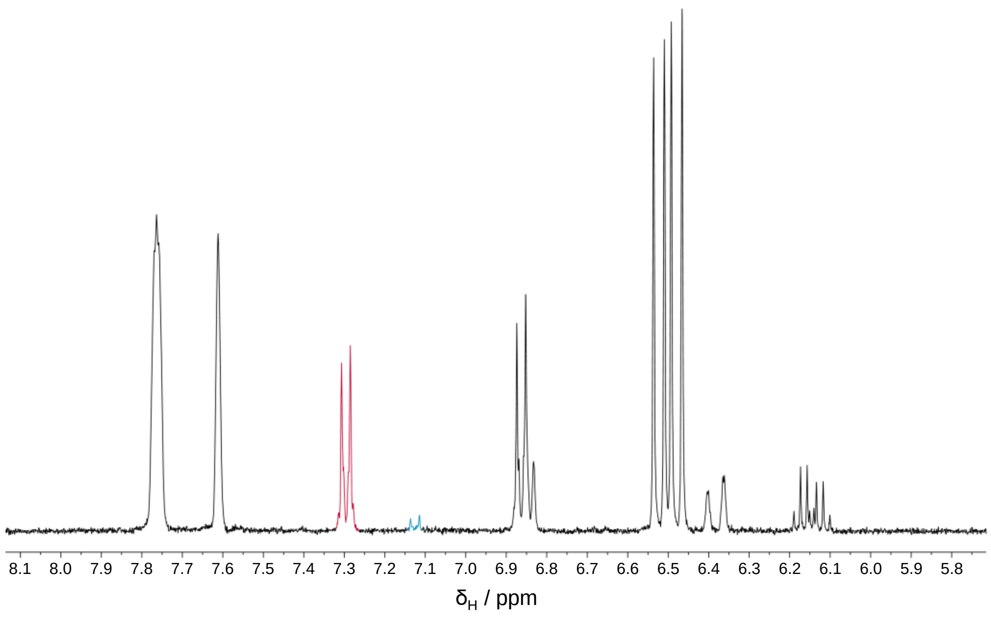

Figure S23: Partial ^{1}H NMR spectra (400 MHz, CD_2Cl_2, 300 K) for the reaction of *trans*-anethole (10 mM) and isoprene (400 mM) in the presence of NaBArF (saturated solution) irradiated with 455 nm light (15 min), demonstrating formation of trace product only. A selected signal of *trans*-anethole is highlighted in red and one of product is highlighted in blue.

3.6.9 ^{1}H NMR spectra of *in situ* irradiated [4+2] cycloadditions

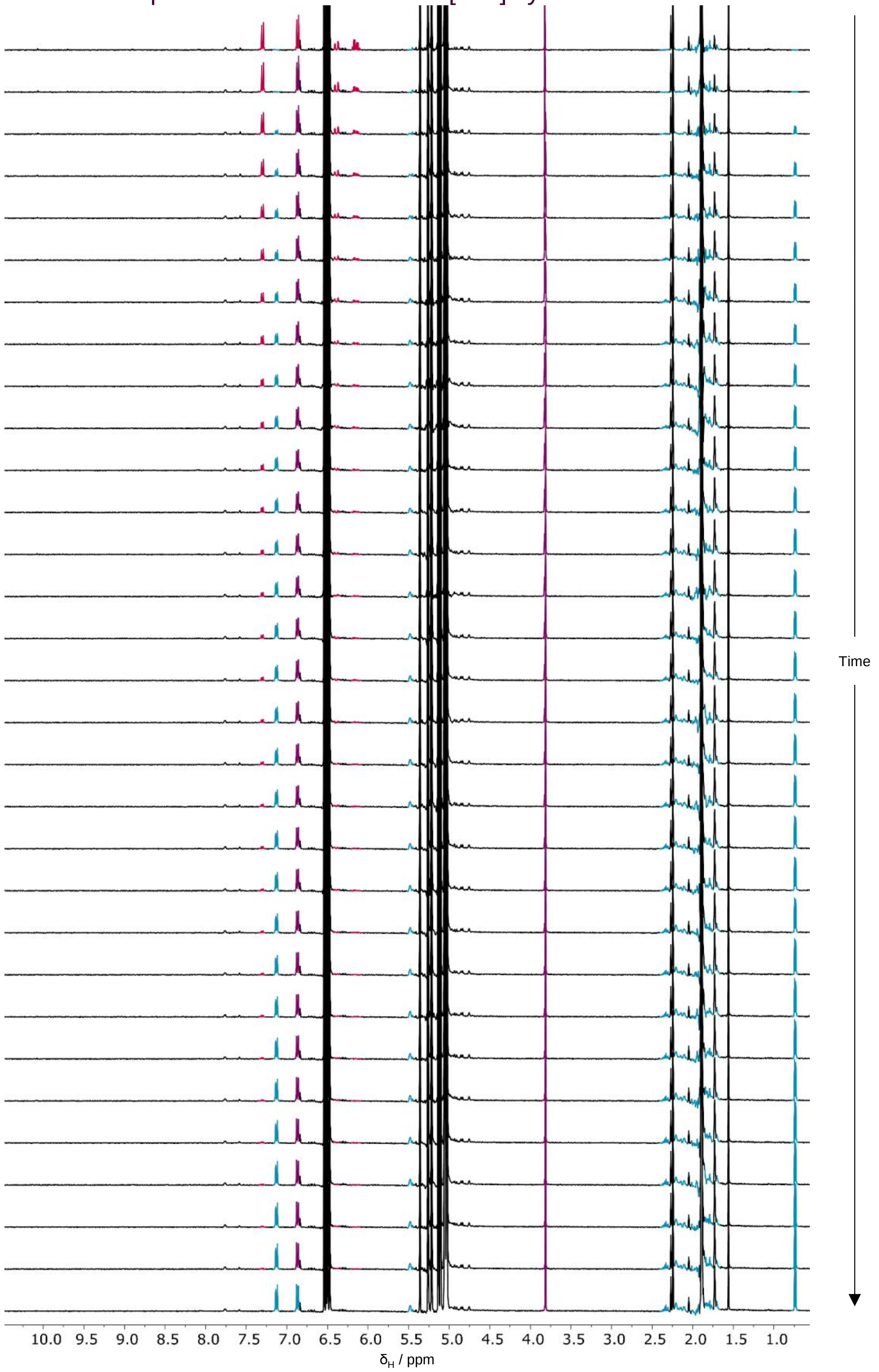

Figure S24: ^{1}H NMR spectra (400 MHz, CD_2Cl_2, 300 K) for the reaction of *trans*-anethole (10 mM) and isoprene (400 mM) in the presence of **C1** (0.1 mM) and **Q1** (0.1 mM) irradiated *in situ* with 455 nm light (5% power). *trans*-Anethole is highlighted in red, product is highlighted in blue, overlapping *trans*-anethole and cycloadduct peak highlighted in purple.

3.7 Measuring Radical Chain Length Using LED-NMR

3.7.1 Calculating the intensity of the LED-NMR light source with ferrioxolate actinometry

All of the below manipulations were conducted in a dark room under red illumination, and the experiment was designed following literature precedent.[7]

Sulfuric acid (14 mL, 17.8 M) was diluted up to 500 mL with H_2O to form a 0.5 M aqueous solution of sulfuric acid. Sulfuric acid (10 mL, 0.5 M) was diluted up to 100 mL with H_2O to form a 0.05 M aqueous solution of sulfuric acid. Potassium ferrioxalate trihydrate (1.85 g, 3.76 mmol) was dissolved in sulfuric acid (25 mL, 0.05 M). Sodium acetate (22.6 g, 277 mmol, 49 eq.) was dissolved in sulfuric acid (100 mL, 0.5M). 1,10-phenanthroline (1.02 g, 5.67 mmol. 1 eq.) was then added.

An aliquot of the potassium ferrioxalate solution (0.28 mL) was added to an amberised NMR tube, into which was then placed the LED-NMR insert. The solution was then illuminated for the required time at 455 nm, at the 5% setting on the LED driver. 10 µL of this solution was then added to an aliquot of the sodium acetate:phenanthroline solution (5 mL) containing a magnetic stirrer bar. The mixture was left to stir for one hour in the dark. A UV-Vis spectrum was then taken of this solution and the absorbance at 510 nm was used to calculate the amount of Fe^{2+} in the illuminated solution, as shown below:

$$[Fe(C_2O_4)_3]^{3-} \xrightarrow{h\nu} Fe^{2+} + 2(C_2O_4^{2-}) + C_2O_4^{-}$$

$$Fe^{2+} + 3(\text{phen}) \longrightarrow [Fe(\text{phen})_3]^{2+}$$

From the Beer-Lambert law:

$$[(\mathrm{Fe(phen)}_3)^{2+}] \text{ in the 5 mL solution} = \frac{\Delta A}{\varepsilon b}$$

Where:

ΔA = absorbance at 510 nm relative to a non-irradiated control sample

ε = extinction coefficient of $[Fe(phen)_3]^{2+}$ at 510 nm (11 100 L cm^{-1} mol^{-1})[7,8]

b = path length of the light through the solution (1 cm)

Thus,

$$n((\mathrm{Fe(phen)}_3)^{2+} \text{ in the 5 mL solution}) = \frac{\Delta A \times 0.005\ L}{1\ cm \times 11100\ Lcm^{-1}mol^{-1}}$$

And the overall n(Fe^{2+} generated in the NMR tube by LED-NMR illumination) = n(($Fe(phen)_3$)$^{2+}$ in the 5 mL solution $\times \frac{280}{10}$.

Plotting this for each data point:

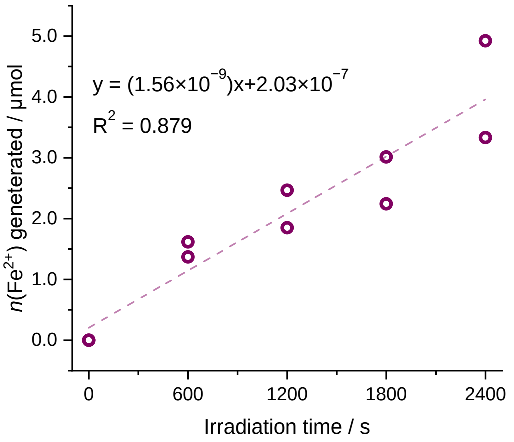

Figure S25: A plot of Fe^{2+} generated as a function of LED irradiation time within the NMR tube.

By least squares fitting the gradient of the graph is calculated to be 1.6 ± 0.02 × 10^{-9} mol s^{-1} of Fe^{2+} generated.

Given that:

Φ($K_3[Fe(C_2O_4)_3]$) at 458 nm = 0.844 (from literature)[8]

(We take this as a reasonable approximate of the value at 455 nm.)

And:

$$\Phi = \frac{n(\text{product})}{n(\text{photons})}$$

Under these conditions:

$$n(\text{photons absorbed by } K_3[\text{Fe}(C_2O_4)_3]) = \frac{n(\text{product})}{\Phi} = \frac{1.6 \times 10^{-9}}{0.844} = 1.8 \pm 0.2 \ \times 10^{-9}\ mol\ s^{-1}$$

However, given the short path length and high wavelength of light used, it is unlikely that the actinometer is absorbing all the light in the system. Thus the actual incident light intensity must be calculated.

From the Beer-Lambert law:

$$n(\text{photons absorbed by } K_3[\text{Fe}(C_2O_4)_3]) = I_0(1 - 10^{-\varepsilon bc})$$

Where:

I_0 = the number of incident photons

ε = extinction coefficient of complex at wavelength of irradiation.

b = path length (measured to be 0.44 mm for our LED-NMR apparatus)

c = concentration of $K_3[Fe)C_2O_4)_3]$ (0.15 M)

From the literature, logε of the $K_3[Fe(C_2O_4)_3]$ at 450 nm is approximately 1.3.[9] Extrapolation of the graph to 455 nm puts this at approximately 1.2 and we take this as a reasonable approximate of the true value. Thus:

$$\varepsilon = 10^{1.2} = 16\ M^{-1}\ cm^{-1}$$

And therefore:

$$\text{I}_0 \text{of the LED} - \text{NMR light source} = \frac{1.8\ \pm 0.2\ \times 10^{-9}}{1 - 10^{-(16\times 0.044\ \times 0.15)}} = 9 \pm 1\ \times 10^{-9}\ \text{mol s}^{-1}$$

3.7.2 Calculating how many incident photons are absorbed by the active catalyst

3.7.2.1 Measurement of extinction coefficient of **Q1⊂C1** and Ru(bpz)$_3$(BArF)$_2$

Stock solutions were prepared using spectrophotometry grade CH_2Cl_2 and standard volumetric glassware. Solutions of the host-guest complex **Q1⊂C1** were prepared in a 1:1.2 ratio to minimise the concentration of unbound quinone. Stock solutions were diluted and transferred to quartz cuvettes with 1 mm pathlength for spectral acquisition.

UV spectra were recorded on Ocean-Optics Flame CCD Spectrometer connected via optical fibre to a DH2000-BALUV lamp. The samples were housed in a thermostatted (20.0°C ± 0.1°C) cuvette holder. Acquisition was controlled by the Kinetic Studio software package (version 5). Data were processed and plotted using Excel (replotted using Origin).

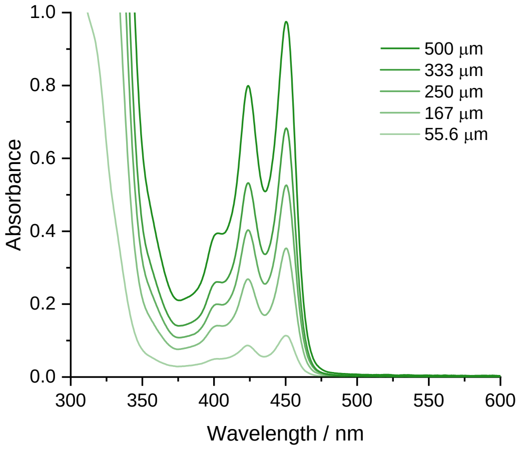

Figure S26: UV-vis absorption spectra for high concentration solutions of **Q1⊂C1** in CH_2Cl_2. l_{max} = 450.51 nm.

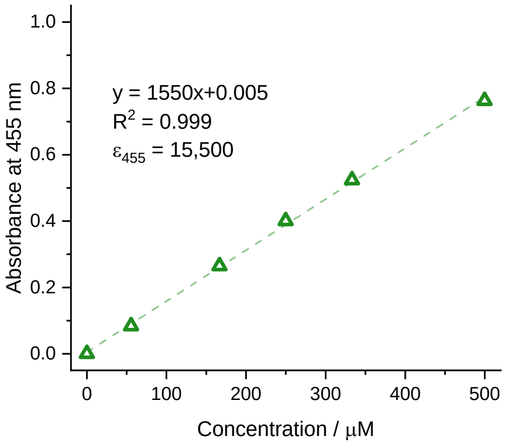

Figure S27: Experimental (points) and simulated (filled line) absorbance at 455 nm of **Q1⊂C1** as a function of concentration. The molar absorptivity (inset) is calculated via linear regression, according to the Beer-Lambert Law.

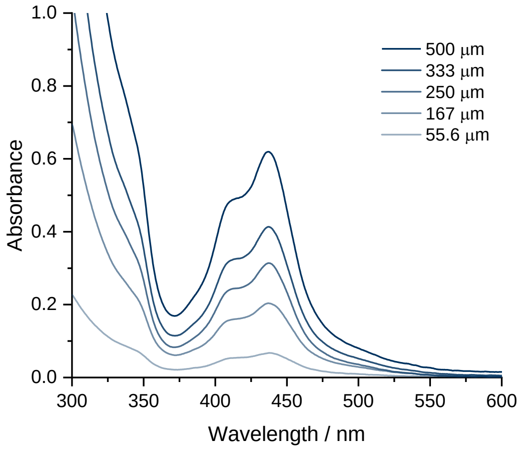

Figure S28: UV-vis absorption spectra for high concentration solutions of $Ru(bpz)_3(BArF)_2$ in CH_2Cl_2. l_{max} = 437.66 nm.

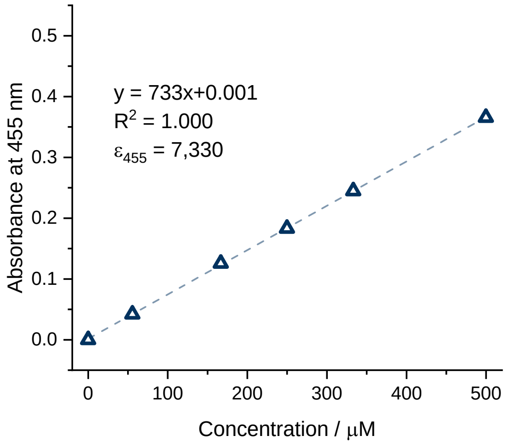

Figure S29: Experimental (points) and simulated (filled line) absorbance at 455 nm of $Ru(bpz)_3(BArF)_2$ as a function of concentration. The molar absorptivity (inset) is calculated via linear regression, according to the Beer-Lambert Law.

3.7.2.2 Calculation from Beer-Lambert law

The number of photons absorbed by a species = $I_0(1 - 10^{-\varepsilon bc})$

Where:

I_0 = number of incident photons

ε = extinction coefficient of complex at 455 nm (calculated above)

b = path length (measured to be 0.44 mm for our LED-NMR apparatus)

c = complex concentration (taken as 0.1 mM)

Thus the amount of photons absorbed by:

Q1⊂C1 = 1.2 ± 0.2 × 10^{-9} mol photons s^{-1} (inside the entire solution volume)

$Ru(bpz)_3(BArF)_2$ = 6.2 ± 0.8 × 10^{-10} mol photons s^{-1} (inside the entire solution volume)

3.7.3 Calculating how much product is produced by the active catalyst

For **Q1⊂C1**:

A line was fitted to the graph of **Q1⊂C1** loading against initial rate. Each data point represents the average of two different runs. By least squares fitting the gradient was determined to be 1.14 ± 0.03 mM s^{-1} per mM catalyst.

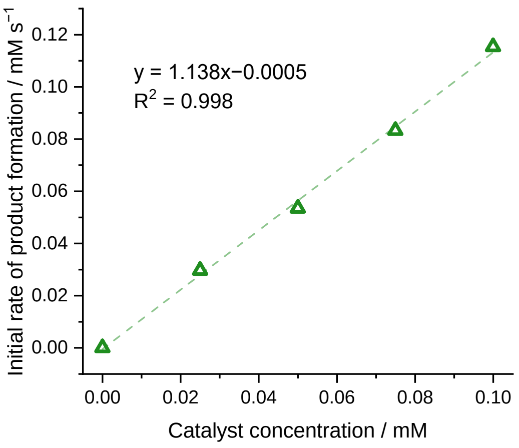

Figure S30: Initial rates of product formation against **Q1⊂C1** concentration.

Thus, within this concentration range, for each unit of **Q1⊂C1** complex present in solution, we generate 1.14 units of product s^{-1}.

Taking a reaction with 0.1 mM catalyst loading as a case study: there is 280 µL of a solution containing 0.1 mM **Q1⊂C1**, *i.e.* 2.8 × 10^{-8} mol of catalyst. Thus, from the above ratio, we produce 3.19 ± 0.09 × 10^{-8} mol product s^{-1}.

For $Ru(bpz)_3(BArF)_2$:

A line was fitted to the graph of $Ru(bpz)_3(BArF)_2$ loading against initial rate. Each data point represents the average of two different runs. By least squares fitting the gradient was determined to be 1.79 ± 0.09 mM s^{-1} per mM catalyst.

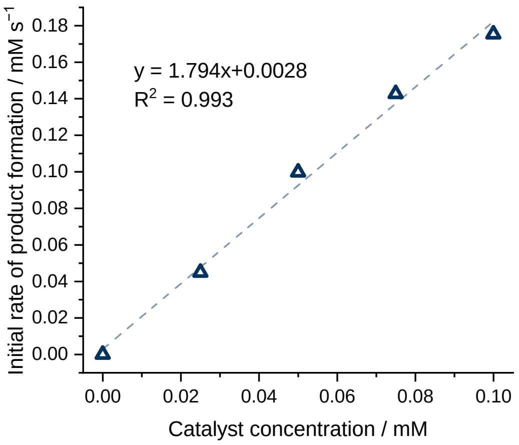

Figure S31: Initial rates against $Ru(bpz)_3(BArF)_2$ loading.

Thus, within this concentration range, for each unit of $Ru(bpz)_3(BArF)_2$ complex present in solution, we generate 1.79 units of product s^{-1}.

Taking a reaction with 0.1 mM catalyst loading as a case study: there is 280 µL of a solution containing 0.1 mM $Ru(bpz)_3(BArF)_2$, *i.e.* 2.8 × 10^{-8} mol of catalyst. Thus, from the above ratio, we produce 5.0 ± 0.2 × 10^{-8} mol product s^{-1}.

3.7.4 Calculation of catalyst quantum yield

For **Q1⊂C1**:

$$\phi = \frac{n(\text{product})}{n(\text{photons})} = \frac{3.19 \times 10^{-8}\, mol\ s^{-1}}{1.2\ \times 10^{-9}\, mol\ s^{-1}} = 25 \pm 4$$

For $Ru(bpz)_3(BArF)_2$:

$$\phi = \frac{n(\text{product})}{n(\text{photons})} = \frac{5.0 \times 10^{-8}\ mol\ s^{-1}}{6.2\ \times 10^{-10}\ mol\ s^{-1}} = 80 \pm 10$$

For the $Ru(bpz)_3(BArF)_2$ complex, this value is approximately double that reported by Cismesia and Yoon.[10] This discrepancy presumably arises from the differences between the two methods used to estimate quantum yield. In this work we estimate it based on an initial rates relationship; in contrast, Cismesia and Yoon use the total yield after a certain amount of time. These two different methods of calculating quantum yield will inevitably result in somewhat different numbers – calculations based on end points will typically provide smaller values as addition of some amount of photons to a reaction that is near or at completion will result in much less (or no) product formation than addition of that amount of photons near the beginning of a reaction. In contrast, calculations based on initial rates trend towards giving a higher value as they use a regime where the maximum proportion of absorbed photons are productively utilised to generate product. The latter is representative of the optimal radical chain length; however, it does not take into account progressive inhibition of the reaction, catalyst decomposition and other more complex mechanistic phenomena.

4 Cage-Quinone Catalyzed 1,3-Cyclohexadiene Cycloaddition Reactions

4.1 General Procedure

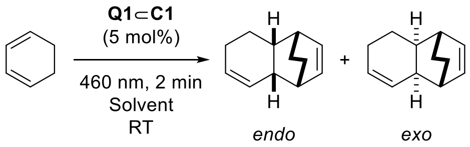

Stock solutions in the required deuterated solvent were prepared for: 2-fluoromesitylene (internal standard, 1.20 mmol/mL), 1,3-cyclohexadiene (2.40 mmol/mL) and **C1** (3.0×10^{-3} mmol/mL). A stock solution in CH_2Cl_2 was prepared for **Q1** (1.0×10^{-3} mmol/mL).

Q1 (0.09 mg, 3.0×10^{-4} mmol, 0.05 eq., 300 µL) was added to an NMR tube. The solvent was removed under a stream of N_2 and the tube was dried *in vacuo* for 1 h. To the NMR tube, deuterated solvent (425 µL), internal standard (0.4 mg, 3.0×10^{-3} mmol, 0.5 eq., 25 µL) and 1,3-cyclohexadiene (0.5 mg, 6.0×10^{-3} mmol, 1.0 eq., 25 µL) were added. The reaction was initiated by addition of **C1** (1.4 mg, 3.0×10^{-4} mmol, 0.05 eq., 100 µL) and blue LED (460 nm) irradiation for 2 min. The reaction was monitored by recording ^{1}H NMR spectra before the addition of **C1** and after the irradiation session. Product yields were calculated by comparing integrals of the starting material and product[s] to that of the internal standard.

The reaction was screened with the following solvents:

- CD_2Cl_2
- CD_3NO_2
- CD_3CN

4.1.1 Additive Experiment

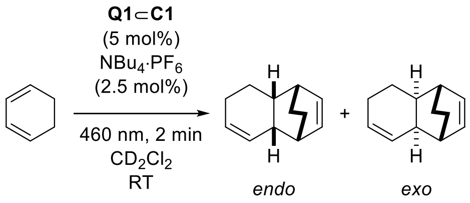

The above procedure for CD_2Cl_2 was modified with the addition of $NBu_4{\cdot}PF_6$ (0.06 mg, 1.5×10^{-4} mmol, 0.025 eq.) prior to collecting the initial ^{1}H NMR spectrum (*i.e.* prior to the addition of **C1**).

4.1.2 Aerated Procedure

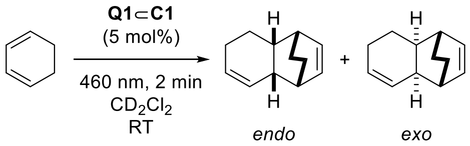

A stock solution in CH_2Cl_2 was prepared for **Q1** (1.3×10^{-3} mmol/mL). **Q1** (0.45 mg, 1.5×10^{-3} mmol, 0.05 eq., 1.1 mL), 2-fluoromesitylene (2.2 µL, 2.1 mg, 3.0×10^{-3} mmol, 0.5 eq.) and 1,3-cyclohexadiene (2.9 µL, 2.4 mg, 3.0×10^{-2} mmol, 1.0 eq.) were placed into a microwave vial containing CH_2Cl_2 (1.9 mL). In the absence of light, **C1** (7.2 mg, 1.5×10^{-3} mmol, 0.05 eq.) was added and the microwave vial was sealed. An aliquot (400 µL) was immediately taken and added to an NMR tube containing CD_2Cl_2 (200 µL), which was kept in the dark to avoid photoreaction. The reaction was irradiated with 460 nm light for 2 min under a constant stream of compressed air. Another aliquot (400 µL) was taken and added to an NMR tube containing CD_2Cl_2 (200 µL). Product yields were calculated by comparing integrals of the starting material and product to that of the internal standard.

4.2 Product Identification

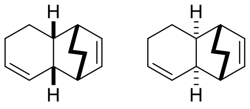

endo *exo*

The product of each NMR scale reaction was identified (and thereby yields calculated) by comparing the spectra to previously reported ^{1}H NMR spectroscopic data.[11]

4.3 ^{1}H NMR spectra of [4+2] catalyzed reactions

4.3.1 CD_2Cl_2

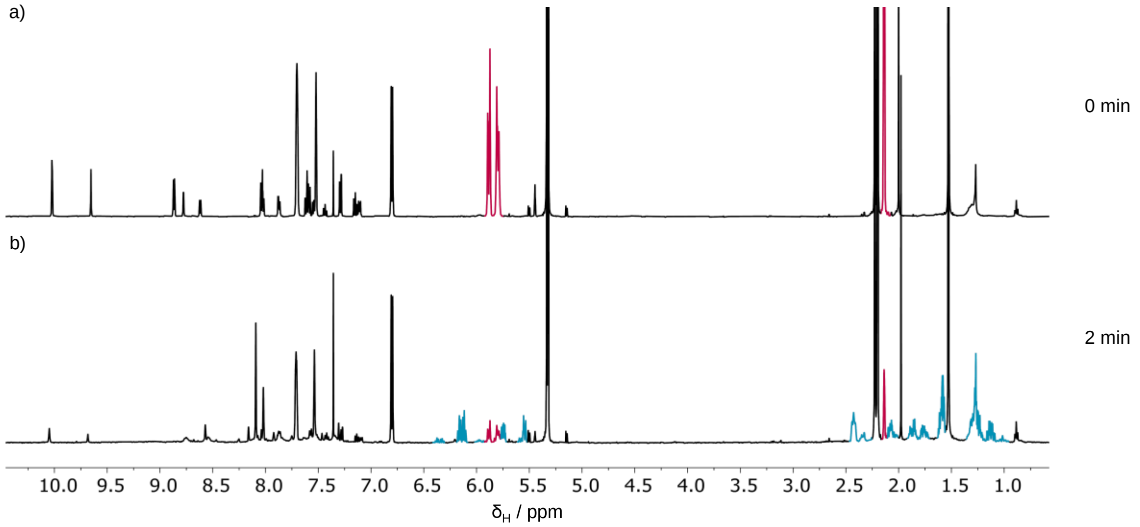

Figure S32: ^{1}H NMR spectra (500 MHz, CD_2Cl_2, 300 K) for the reaction of 1,3-cyclohexadiene (10 mM) in the presence of **C1** (0.5 mM) and **Q1** (0.5 mM) after irradiation with 460 nm light, t = 2 min. (a) 1,3-cyclohexadiene and **Q1** at t = 0 min. (b) 1,3-cyclohexadiene, **C1** and **Q1** after irradiation with 460 nm light, t = 2 min. 1,3-cyclohexadiene is highlighted in red, cycloadducts are highlighted in blue.

4.3.2 CD_3NO_2

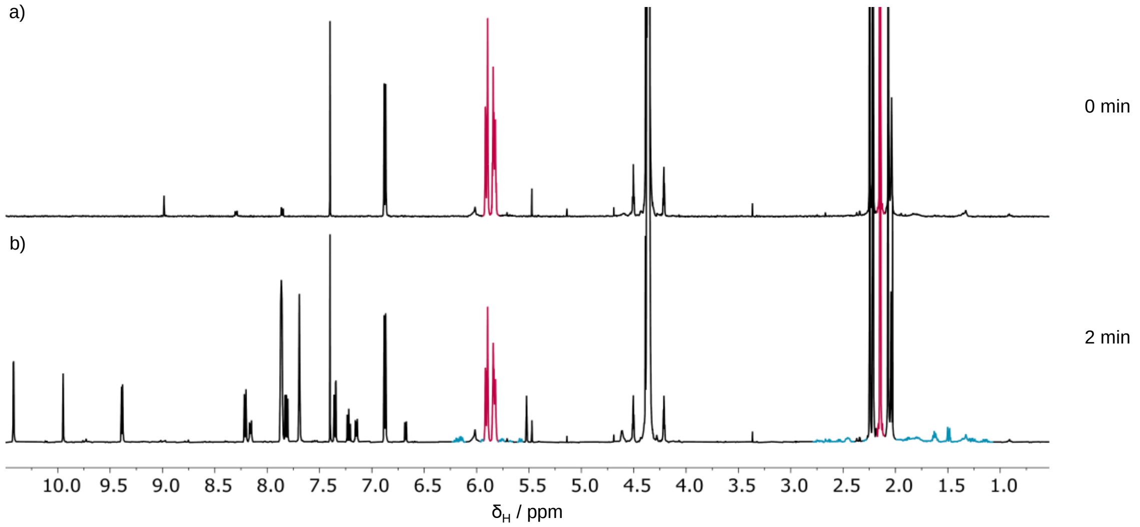

Figure S33: ^{1}H NMR spectra (500 MHz, CD_3NO_2, 300 K) for the reaction of 1,3-cyclohexadiene (10 mM) in the presence of **C1** (0.5 mM) and **Q1** (0.5 mM) after irradiation with blue LEDs (460 nm), t = 2 min. (a) 1,3-cyclohexadiene and **Q1** at t = 0 min. (b) 1,3-cyclohexadiene, **C1** and **Q1** after irradiation with 460 nm light, t = 2 min. 1,3-cyclohexadiene is highlighted in red, cycloadducts are highlighted in blue.

4.3.3 CD_3CN

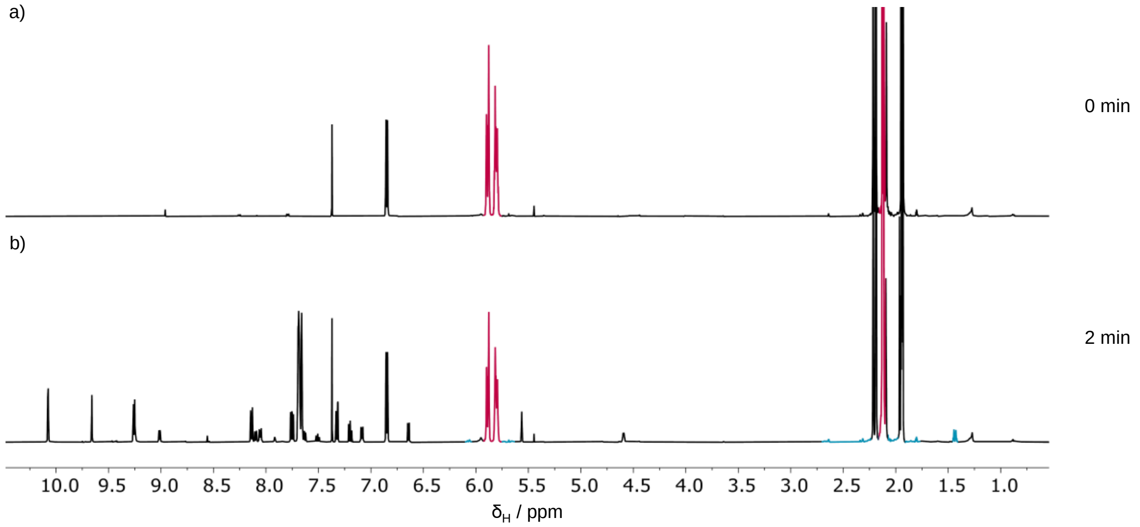

Figure S34: ^1H NMR spectra (500 MHz, CD_3CN, 300 K) for the reaction of 1,3-cyclohexadiene (10 mM) in the presence of **C1** (0.5 mM) and **Q1** (0.5 mM) after irradiation with blue LEDs (460 nm), t = 2 min. (a) 1,3-cyclohexadiene and **Q1** at t = 0 min. (b) 1,3-cyclohexadiene, **C1** and **Q1** after irradiation with 460 nm light, t = 2 min. 1,3-cyclohexadiene is highlighted in red, cycloadducts are highlighted in blue.

4.3.4 CD_2Cl_2 + $NBu_4{\cdot}PF_6$

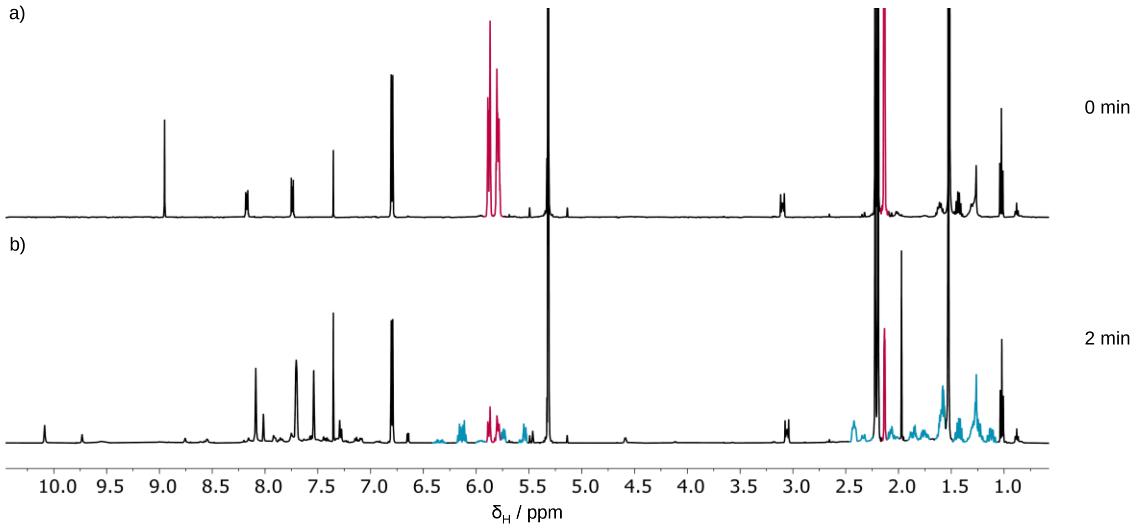

Figure S35: ^1H NMR spectra (500 MHz, CD_2Cl_2, 300 K) for the reaction of 1,3-cyclohexadiene (10 mM) in the presence of **C1** (0.5 mM), **Q1** (0.5 mM) and $NBu_4{\cdot}PF_6$ (2.5 mol%) after irradiation with blue LEDs (460 nm), t = 2 min. (a) 1,3-cyclohexadiene, **Q1** and $NBu_4{\cdot}PF_6$ at t = 0 min. (b) 1,3-cyclohexadiene, **C1**, **Q1** and $NBu_4{\cdot}PF_6$ after irradiation with 460 nm light, t = 2 min. 1,3-cyclohexadiene is highlighted in red, cycloadducts are highlighted in blue.

4.3.5 CD_2Cl_2 + air

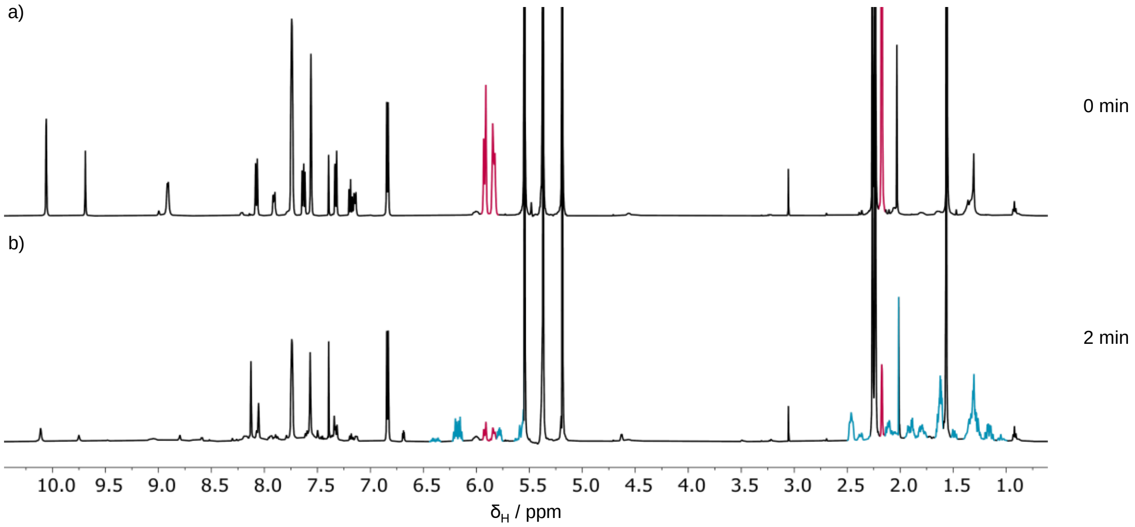

Figure S36: ^{1}H NMR spectra (500 MHz, CD_2Cl_2/CH_2Cl_2, 300 K, with solvent suppression) for the aerated reaction of 1,3-cyclohexadiene (10 mM) in the presence of **C1** (0.5 mM) and **Q1** (0.5 mM) after irradiation with blue LEDs (460 nm), t = 2 min. (a) 1,3-cyclohexadiene and **Q1** at t = 0 min. (b) 1,3-cyclohexadiene, **C1** and **Q1** after irradiation with 460 nm light, t = 2 min. 1,3-cyclohexadiene is highlighted in red, cycloadducts are highlighted in blue.

4.4 *In situ* Kinetic Monitoring with LED-NMR

4.4.1 General Procedure

Stock solutions in CD_2Cl_2 were prepared for: 2-fluoromesitylene (internal standard, 100 mM), 1,3-cyclohexadiene (80 mM), **C1** (5 mM) and $Ru(bpz)_3(BArF)_2$ (5 mM). A stock solution in CH_2Cl_2 was prepared for **Q1** (0.5 mM) and the appropriate amount was measured into a 1 mL volumetric flask. The CH_2Cl_2 was then evaporated and the appropriate volumes of all stock solutions were measured into the flask, with the remaining volume then being made up to 1 mL. The solution was thoroughly homogenised before a 280 µL aliquot was taken for each *in situ* monitoring experiment.

4.4.2 Variation of **Q1⊂C1** concentration

Five reactions were prepared with varying concentrations of **Q1⊂C1** (0.25–0.063mM). The effect of varying **Q1⊂C1** loading on rate of consumption of cyclohexadiene (Figure S37) and formation of *endo*-dimer (Figure S38). Each co-plotted separately for ease of visualisation.

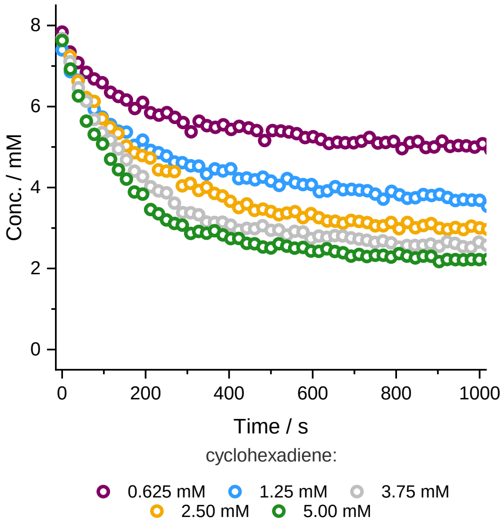

Figure S37: Kinetic traces of consumption of starting cyclohexadiene (8 mM) at varying loadings of **Q1⊂C1** (0.25–0.063 mM). Irradiation is at 5% LED power at 455 nm.

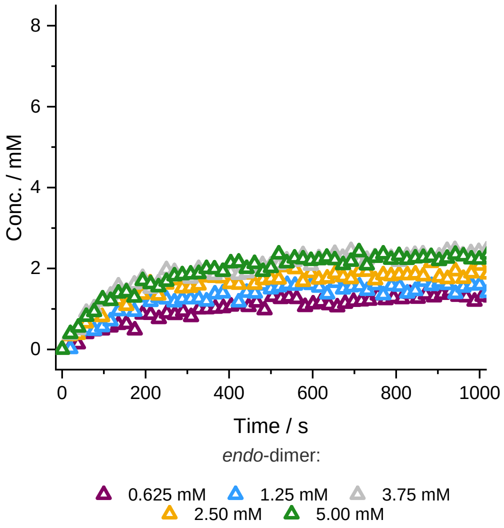

Figure S38: Kinetic traces of formation of *endo*-dimer at varying loadings of **Q1⊂C1**. Irradiation is at 5% LED power at 455 nm.

4.4.3 Variation of light intensity

Two identical solution aliquots were irradiated at 455 nm, at 5% and 100% of the maximum LED intensity. It was observed that raising the light intensity raised the reaction rate, but did not impact on the overall kinetic profile.

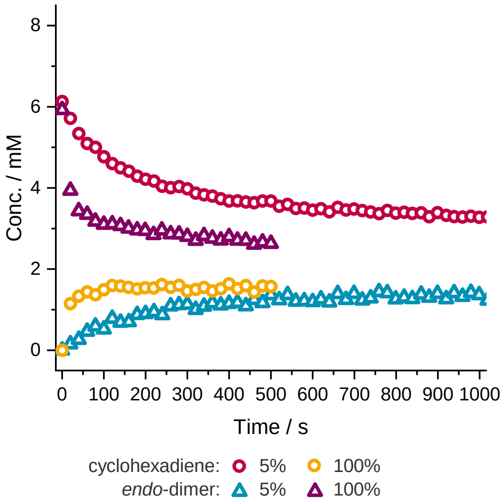

Figure S39: Two overlaid kinetic profiles of the conversion of cyclohexadiene (circles) to *endo*-dimer (triangles) run at two different light intensities.

4.4.4 Variation of $Ru(bpz)_3(BArF)_2$ concentration

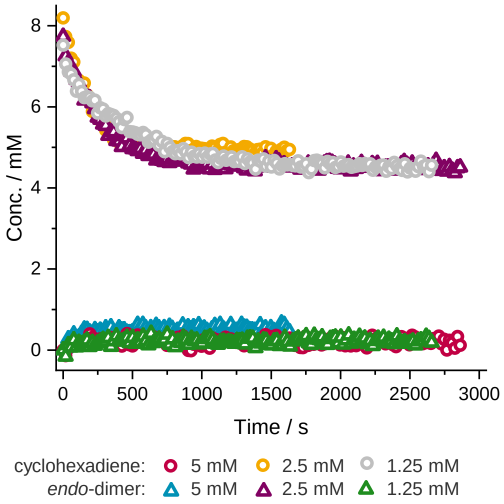

Figure S40: Overlaid kinetic profiles of three otherwise-identical reactions converting cyclohexadiene (circles) to *endo*-dimer (triangles), run at various loadings of $Ru(bpz)_3(BArF)_2$.

4.4.5 Addition of NaBArF to the reactions

Reaction solutions were prepared as per the general procedure discussed in Section 4.4.1. In the instances where NaBArF was added, the fully-assembled solution was saturated with NaBArF prior to irradiation.

4.4.5.1 Reaction using **Q1⊂C1** as catalyst

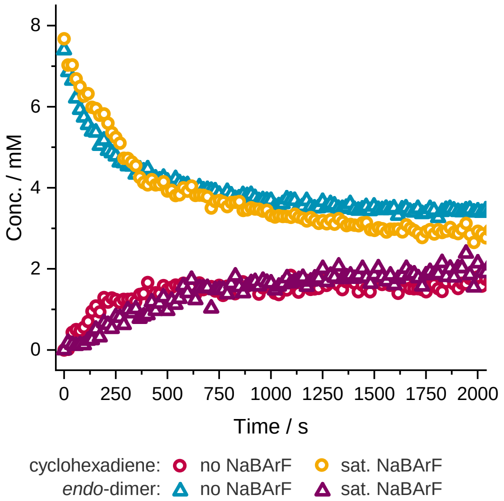

Figure S41: Overlaid kinetic profiles of two otherwise-identical reactions converting cyclohexadiene (circles) to *endo*-dimer (triangles) using 1.25% loading of **Q1⊂C1**, where one reaction solution has been saturated with NaBArF prior to irradiation.

4.4.5.2 Reaction using $Ru(bpz)_3(BArF)_2$ as catalyst

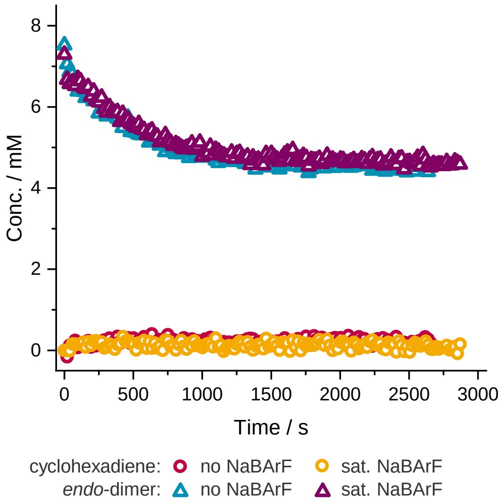

Figure S42: Overlaid kinetic profiles of two otherwise-identical reactions converting cyclohexadiene (circles) to *endo*-dimer (triangles) using 1.25% loading of $Ru(bpz)_3(BArF)_2$, where one reaction solution has been saturated with NaBArF prior to irradiation.

4.4.6 Reaction using only **Q1** and NaBArF

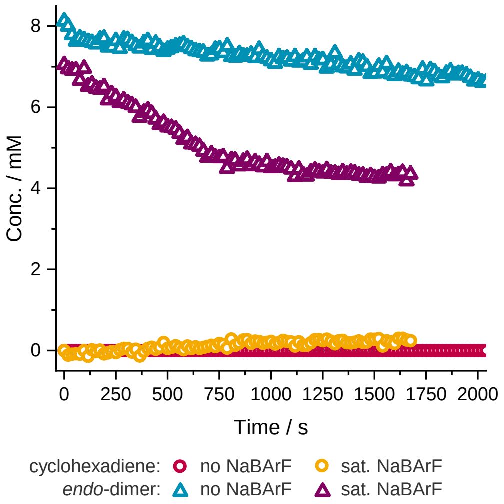

Figure S43: Overlaid kinetic profiles of irradiation of a solution with 5 mol% **Q1** as the photocatalyst. The NMR signal corresponding to cyclohexadiene is represented as circles, the NMR signal corresponding to *endo*-dimer as triangles. No NMR signal corresponding to the *exo*-dimer is observed. One solution is additionally saturated with NaBArF.

4.4.7 Calculation of reaction quantum yield, using **Q1⊂C1** as the catalyst

The same procedure as in Section 3.7 was followed, using the previously measured light intensity and **Q1⊂C1** extinction coefficient. It was previously shown that 0.1 mM catalyst will absorb 1.3 × 10^{-9} mol photons per second.

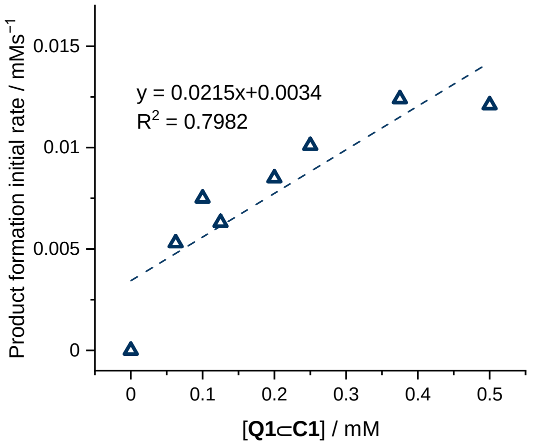

Figure S44: Initial rates of product formation against **Q1⊂C1** concentration.

A line was fitted to the graph of **Q1⊂C1** loading against initial rate of product formation. By least squares fitting the gradient was determined to be 0.022 ± 0.004 mM s^{-1} per mM catalyst.

Thus, within this concentration range, for each unit of **Q1⊂C1** complex present in solution, we generate 0.022 units of product s^{-1}.

Taking a reaction with 0.1 mM catalyst loading as a case study: there is 280 µL of a solution containing 0.1 mM **Q1⊂C1**, *i.e.* 2.8 × 10^{-8} mol of catalyst. Thus, from the above ratio, we produce 6 ± 1 × 10^{-10} mol product s^{-1}.

$$\phi = \frac{n(\text{product})}{n(\text{photons})} = \frac{6 \times 10^{-10}\ mol\ s^{-1}}{1.3\ \times 10^{-9}\ mol\ s^{-1}} = 0.5 \pm 0.2$$

Individual analysis of each reaction using the amount of product formed and photons absorbed after a certain time gave values in line with this result.

4.4.8 Calculation of max TON for cage and Ru catalysed reactions

Turnover number (TON) was calculated as:

$$\mathrm{TON} = \frac{\text{moles product}}{\text{moles catalyst}}$$

For each reaction, this was calculated for ten individual data points after the reaction had plateaued.

Averaging this value across four different reactions catalysed by **Q1⊂C1** yielded a value of 8 ± 1.

Averaging this value across two different reactions catalysed by $Ru(bpy)_3(BArF)_2$ yielded a value of 0.9 ± 0.3.

5 Cage-Quinone Catalyzed Aza-Henry Reaction

5.1 Procedure

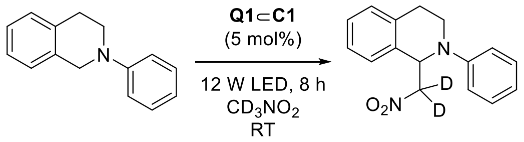

Stock solutions in CD_3NO_2 were prepared for: 2-fluoromesitylene (internal standard, 1.20 mmol/mL), *N*-phenyl-1,2,3,4-tetrahydroisoquinoline (2.40 mmol/mL) and **C1** (3.0×10^{-3} mmol/mL). A stock solution in CH_2Cl_2 was prepared for **Q1** (1.0×10^{-3} mmol/mL).

Q1 (0.09 mg, 3.0×10^{-4} mmol, 0.05 eq., 300 µL) was added to an NMR tube. The solvent was removed under a stream of N_2 and the tube was dried *in vacuo* for 1 h. To the NMR tube, CD_3NO_2 (450 µL), internal standard (0.4 mg, 3.0×10^{-3} mmol, 0.5 eq., 25 µL) **C1** (1.4 mg, 3.0×10^{-4} mmol, 0.05 eq., 100 µL and *N*-phenyl-1,2,3,4-tetrahydroisoquinoline (1.3 mg, 6.0×10^{-3} mmol, 1.0 eq., 25 µL). The reaction was run under white LED (12 W) irradiation for a total time of 8 h (4 × 2 h). The reaction was monitored by recording ^{1}H NMR spectra before irradiation and after each irradiation session. Product yields were calculated by comparing integrals of the starting material and product to that of the internal standard.

5.2 Product Identification

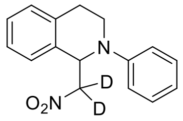

The product of the NMR scale reaction was identified (and thereby yields calculated) by comparing the spectrum to previously reported ^{1}H NMR spectroscopic data (in CD_3NO_2).[12]

As a deuterated substrate was used, the product was characterized following isolation:

The solvent was removed *in vacuo*. The residues were dissolved in hexanes, filtered through a 17 mm PTFE 0.45 µm syringe filter, and the catalyst removed using preparative TLC (hexanes/EtOAc, 5:1), to give the product as a yellow oil. The product was identified by comparing the spectrum to previously reported ^{1}H NMR spectroscopic data (in $CDCl_3$).[13] **^{1}H NMR (500 MHz, $CDCl_3$) δ_H:** 7.32 – 7.11 (m, 6H), 7.00 – 6.96 (m, 2H), 6.85 (tt, *J* = 7.3, 1.0 Hz, 1H), 5.55 – 5.54 (m, 1H), 3.70 – 3.59 (m, 2H), 3.09 (ddd, *J* = 16.6, 9.0, 5.9 Hz, 1H), 2.80 (dt, *J* = 16.6, 4.9 Hz, 1H). **^{13}C NMR (126 MHz, $CDCl_3$) δ_C:** 148.6, 135.4, 133.1, 129.7, 129.3, 128.3, 127.2, 126.9, 119.6, 115.3, 60.5, 57.8, 42.3, 26.7. **m/z** (ESI) 208.1124 ($[M\text{-}CD_2NO_2]^+$, 100%) and 271.1417 ($[M+H]^+$, 100%).

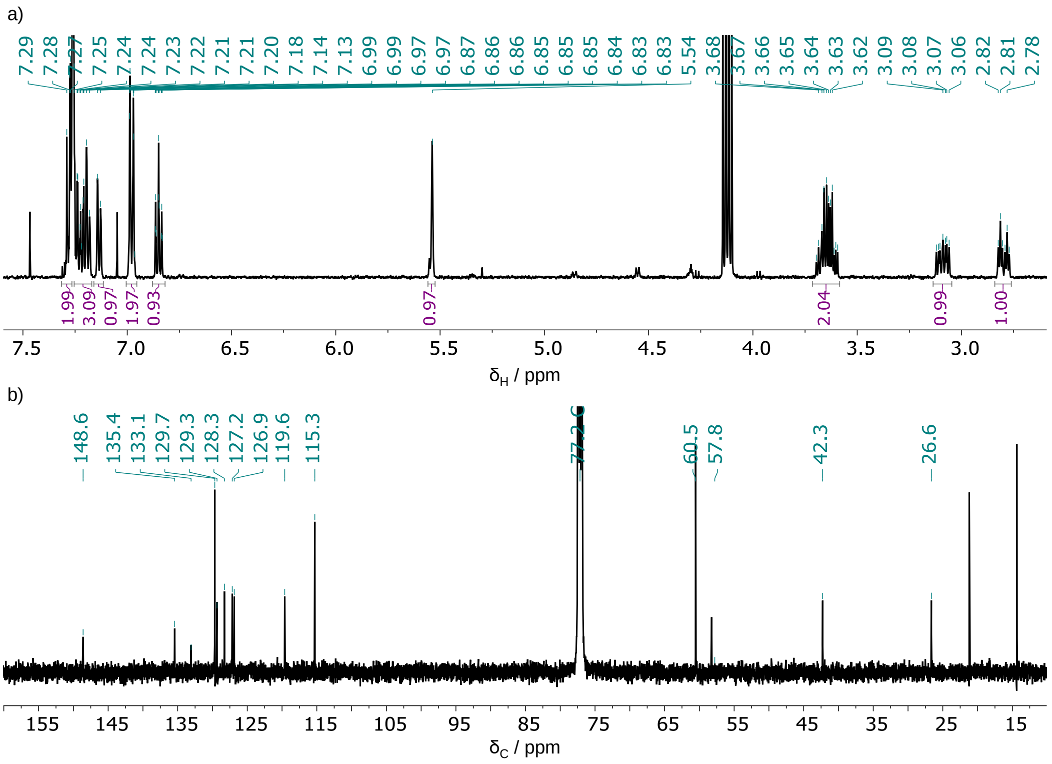

Figure S45: (a) ^{1}H NMR spectrum (500 MHz, $CDCl_3$, 300 K) and (b) ^{13}C NMR spectrum (126 MHz, $CDCl_3$, 300 K) for the cycloadduct isolated from the reaction of *N*-phenyl-1,2,3,4-tetrahydroisoquinoline and nitromethane in the presence of **C1** and **Q1** and white light.

5.3 ^{1}H NMR spectra of the aza-Henry reaction

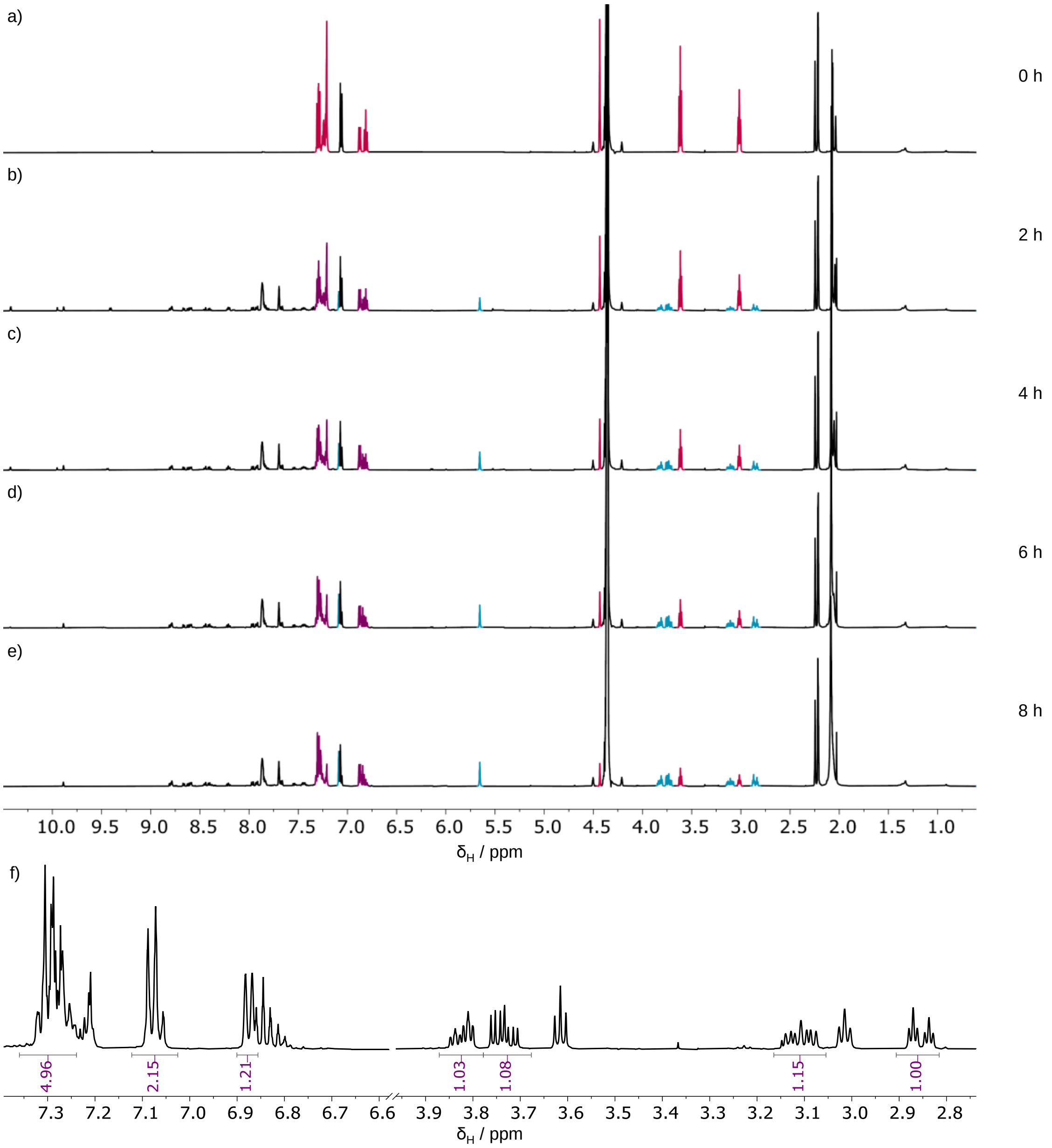

Figure S46: ^{1}H NMR spectra (500 MHz, CD_3NO_2, 300 K) for the reaction of *N*-phenyl-1,2,3,4-tetrahydroisoquinoline (10 mM) and CD_3NO_2 in the presence of **C1** (0.5 mM) and **Q1** (0.5 mM) under white LED (12 W) irradiation: (a) t = 0 h; (b) t = 2 h; (c) t = 4 h; (d) t = 6 h; (e) t = 8 h. (f) Partial ^{1}H NMR spectrum of (e) highlighting product peaks. *N*-Phenyl-1,2,3,4-tetrahydroisoquinoline is highlighted in red, product is highlighted in blue, overlapping *N*-phenyl-1,2,3,4-tetrahydroisoquinoline and product peaks are highlighted in purple.

6 UV-Vis Experiments

6.1 General Procedure

For each reaction, **C1** (0.5 mM) was added to a solution of quinone (0.5 mM) in CH_2Cl_2, taking a spectrum before and after addition of **C1**.

6.2 UV/Vis spectra

6.2.1 Q1

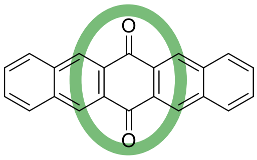

Q1⊂C1

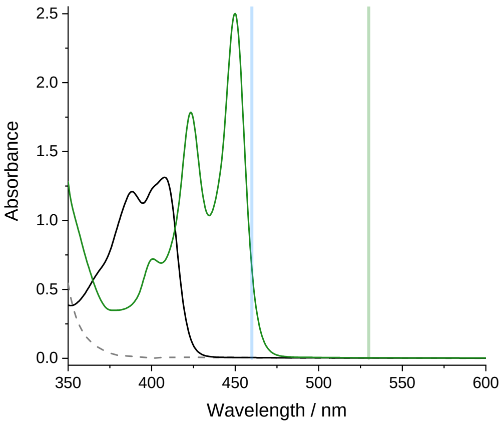

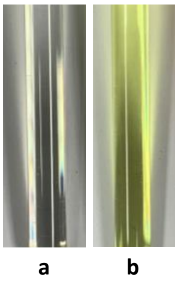

Figure S47: UV-vis spectra (CH_2Cl_2, 298 K) of (i) black line = **Q1** (0.5 mM); (ii) grey dashed line = **C1** (0.5 mM); (iii) green line = **Q1** (0.5 mM) and **C1** (0.5 mM). Vertical blue line at 460 nm, vertical green line at 530 nm. *Picture:* (a) **Q1** (0.5 mM) in CD_2Cl_2; (b) **Q1** (0.5 mM) and **C1** (0.5 mM) in CD_2Cl_2.

6.2.2 Q2

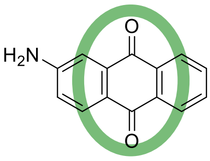

Q2⊂C1

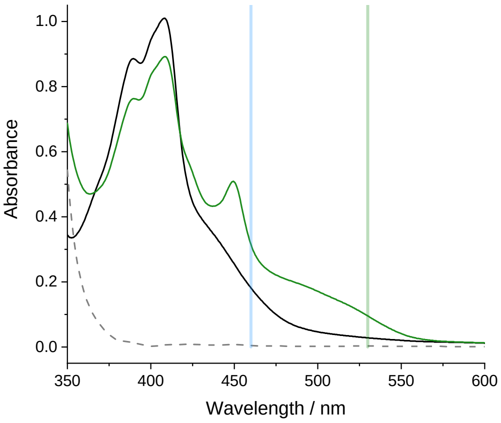

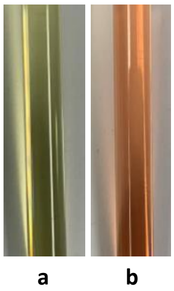

Figure S48: UV-vis spectra (CH_2Cl_2, 298 K) of (i) black line = **Q2** (0.5 mM); (ii) grey dashed line = **C1** (0.5 mM); (iii) green line = **Q2** (0.5 mM) and **C1** (0.5 mM). Vertical blue line at 460 nm, vertical green line at 530 nm. *Picture:* (a) **Q2** (0.5 mM) in CD_2Cl_2; (b) **Q2** (0.5 mM) and **C1** (0.5 mM) in CD_2Cl_2.

6.2.3 **Q1** and NaBArF

A solution was prepared of **Q1** (0.1 mM) in CH_2Cl_2. A UV-Vis spectrum was recorded. The solution was then saturated with NaBArF and another spectrum was recorded.

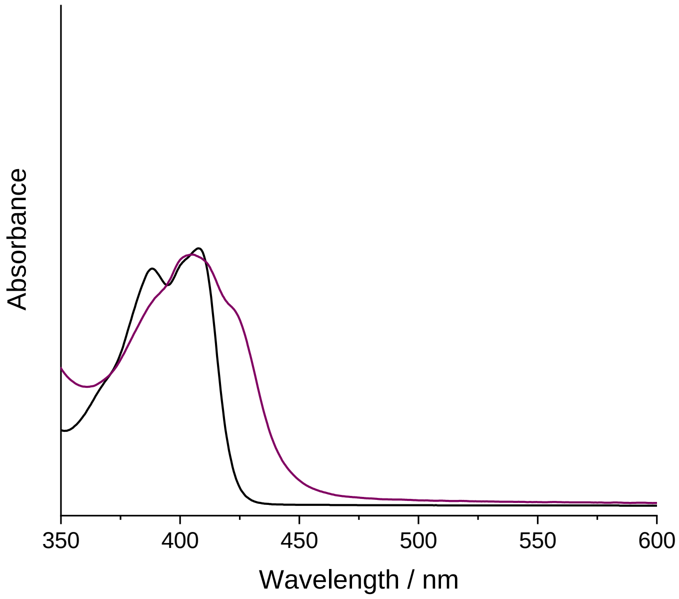

Figure S49: UV-vis spectra (CH_2Cl_2, 298 K) of (i) black line = **Q1** (0.5 mM); (ii) purple line = **Q1** (0.5 mM) in sat. NaBArF.

From this spectrum, the extinction coefficient of **Q1** in a saturated CH_2Cl_2 solution of NaBArF was estimated at 874 $M^{-1}cm^{-1}$

7 References

1 a) C. Feldmeier, H. Bartling, E. Riedle and R. M. Gschwind, *J. Magn. Reson.*, 2013, **232**, 39–44. b) Y. Ben-Tal and G. C. Lloyd-Jones. *J. Am. Chem. Soc*., 2022, **144**, 15372–15382.

2 D. P. August, G. S. Nichol and P. J. Lusby, *Angew. Chem., Int. Ed.*, 2016, **55**, 15022–15026.

3 D. P. Hari and B. König, *Org. Lett.*, 2011, **13**, 3852–3855.

4 L. Bettucci, C. Bianchini, J. Filippi, A. Lavacchi and W. Oberhauser, *Eur. J. Inorg. Chem.*, 2011, **06**, 1797–1805.

5 S. Lies, *Org. Synth.*, 2016, **93**, 178–199.

6 S. M. Stevenson, M. P. Shores and E. M. Ferreira, *Angew. Chem., Int. Ed.*, 2015, **54**, 6506–6510.

7 Y. Ji, D. A. DiRocco, C. M. Hong, M. K. Wismer and M. Reibarkh, *Org. Lett.*, 2018, **20**, 2156–2159.

8 J. N. Demas, W. D. Bowman, E. F. Zalewski and R. A. Velapoldi, *J. Phys. Chem.*, 1981, **85**, 2766–2771.

9 H. Heath, *Proc. R. Soc. London. Ser. A. Math. Phys. Sci.*, 1956, **235**, 518–536.

10 M. A. Cismesia and T. P. Yoon, *Chem. Sci.*, 2015, **6**, 5426–5434.

11 K. T. Lorenz and N. L. Bauld, *J. Am. Chem. Soc.*, 1987, **109**, 1157–1160.

12 H. Bartling, A. Eisenhofer, B. Konig and R. M. Gschwind, *J. Am. Chem. Soc.*, 2016, **138**, 11860–11871.

13 A. S.-K. Tsang, A. S. K. Hashmi, P. Comba, M. Kerscher, B. Chan and M. H. Todd, *Chem. - Eur. J.*, 2017, **23**, 9313–9318.
